# Inhibition of phototrophic iron oxidation by nitric oxide in ferruginous environments

**DOI:** 10.1038/s41561-024-01560-9

**Published:** 2024-10-04

**Authors:** Verena Nikeleit, Adrian Mellage, Giorgio Bianchini, Lea Sauter, Steffen Buessecker, Stefanie Gotterbarm, Manuel Schad, Kurt Konhauser, Aubrey L. Zerkle, Patricia Sánchez-Baracaldo, Andreas Kappler, Casey Bryce

**Affiliations:** 1 Geomicrobiology, University of Tübingen, Tübingen, Germany; 2 NORCE Norwegian Research Center, Bergen, Norway; 3 Civil and Environmental Engineering, University of Kassel, Kassel, Germany; 4 School of Geographical Sciences, University of Bristol, Bristol, UK; 5 Department of Earth System Science, Stanford University, Stanford, CA USA; 6 School of Life Sciences, Arizona State University, Tempe, AZ USA; 7 Department of Earth and Atmospheric Sciences, University of Alberta, Edmonton, Alberta Canada; 8 Blue Marble Space Institute of Science, Seattle, WA USA; 9 Cluster of Excellence EXC 2124: Controlling Microbes to Fight Infections, Tübingen, Germany; 10 School of Earth Sciences, University of Bristol, Bristol, UK

**Keywords:** Element cycles, Geochemistry

## Abstract

Anoxygenic phototrophic Fe(II) oxidizers (photoferrotrophs) are thought to have thrived in Earth’s ancient ferruginous oceans and played a primary role in the precipitation of Archaean and Palaeoproterozoic (3.8–1.85-billion-year-old) banded iron formations (BIFs). The end of BIF deposition by photoferrotrophs has been interpreted as the result of a deepening of water-column oxygenation below the photic zone, concomitant with the proliferation of cyanobacteria. However, photoferrotrophs may have experienced competition from other anaerobic Fe(II)-oxidizing microorganisms, altering the formation mechanism of BIFs. Here we utilize microbial incubations to show that nitrate-reducing Fe(II) oxidizers metabolically outcompete photoferrotrophs for dissolved Fe(II). Moreover, both experiments and numerical modelling show that the nitrate-reducing Fe(II) oxidizers inhibit photoferrotrophy via the production of toxic intermediates. Four different photoferrotrophs, representing both green sulfur and purple non-sulfur bacteria, are susceptible to this toxic effect despite having genomic capabilities for nitric oxide detoxification. Indeed, despite nitric oxide detoxification mechanisms being ubiquitous in some groups of phototrophs at the genomic level (for example, Chlorobi and Cyanobacteria) it is likely that they would still be affected. We suggest that the production of reactive nitrogen species during nitrate-reducing Fe(II) oxidation in ferruginous environments may have inhibited the activity of photoferrotrophs in the ancient oceans and thus impeded their role in the precipitation of BIFs.

## Abstract (web summary)

Banded iron formation deposition by photoferrotrophic organisms in the early Earth’s oceans may have been inhibited by competition for iron and toxicity from nitrate-reducing microorganisms, according to a microbial incubation and numerical modelling study.

## Main

Anoxygenic photoautotrophic Fe(II)-oxidizing bacteria, or ‘photoferrotrophs’ (whose action is shown in equation (1)), are thought to have thrived in Earth’s oceans before the rise in O_2_ and contributed to the deposition of banded iron formations (BIFs)^1–3^. As O_2_ began to rise, these microbes would have seen their habitats shrink, yet they are still thought to have been capable of out-competing abiotic Fe(II) oxidation by O_2_ or respiration by microaerophilic Fe(II) oxidizers while the oxycline remained in the photic zone^4^, that is, when photons (*hν*) could reach deeper anoxic waters.

1 $${{{\mathrm{HCO}}}}_{3}^{-}+4{{{\mathrm{Fe}}}}^{2+}+10{{\mathrm{H}}}_{2}{\mathrm{O}}\mathop{\to }\limits^{h\nu }\left\langle {{{\mathrm{CH}}}}_{2}{\mathrm{O}}\right\rangle +4{{\mathrm{Fe}}}\left({{\mathrm{OH}}}\right)_{3}+7{{\mathrm{H}}}^{+}$$

However, the rise in O_2_ would also have shifted the balance of other biogeochemical cycles towards more oxidized states. Specifically, an increased abundance of nitrate (NO_3_^−^) led to pervasive denitrification in stratified water columns during the Great Oxidation Event (GOE)^5^ that began circa 2.45 billion years ago (Ga). Even before the GOE, evidence exists for transient, localized cycling of oxidized nitrogen species associated with areas of locally elevated O_2_ (oxygen oases) from 2.7 Ga (ref. ^6^) (with some estimates as early as 2.9 Ga (ref. ^7^)), although there is some debate regarding whether the δ^15^N record could reflect other nitrogen cycling processes independent of oxidative nitrogen cycling before 2.3 Ga (refs. ^5,6,8–10^). The input of NO_*x*_ (nitrogen oxides) from atmospheric photochemical reactions would also have supplied oxidized nitrogen species to the oceans as far back as the Hadean^11–13^; with potential for nitrite (NO_2_^−^) to be abiotically reduced to nitric oxide (NO) and nitrous oxide (N_2_O) via abiotic mineral-catalysed reactions^14^.

In modern anoxic environments that contain both Fe(II) and NO_3_^−^, NO_3_^−^ reduction coupled with Fe(II) oxidation (equation (2)) is widespread^15^. During this process, Fe(II) oxidation can be enzymatically driven^16,17^ and/or occur abiotically via chemodenitrification^18^, which is catalysed by reactive nitrogen intermediates, such as NO_2_^−^ and NO, produced during the enzymatic reduction of NO_3_^−^. In modern environments, such as sediments^19–22^ and stratified water columns^23^, both nitrate-reducing and phototrophic Fe(II) oxidizers have been found together. Oxidation of Fe(II) coupled with NO_3_^−^ reduction could, therefore, compete with photoferrotrophs for Fe(II) in regions where NO_3_^−^ and light were available but O_2_ was absent.

2 $$10{{{\mathrm{Fe}}}}^{2+}+2{{{\mathrm{NO}}}}_{3}^{-}+24{{\mathrm{H}}}_{2}{\mathrm{O}}\to 10{{\mathrm{Fe}}}\left({{\mathrm{OH}}}\right)_{3}+{{\mathrm{N}}}_{2}+18{{\mathrm{H}}}^{+}$$

## Nitrate-reducing Fe(II) oxidation inhibits photoferrotrophy

**Fig. 1:**
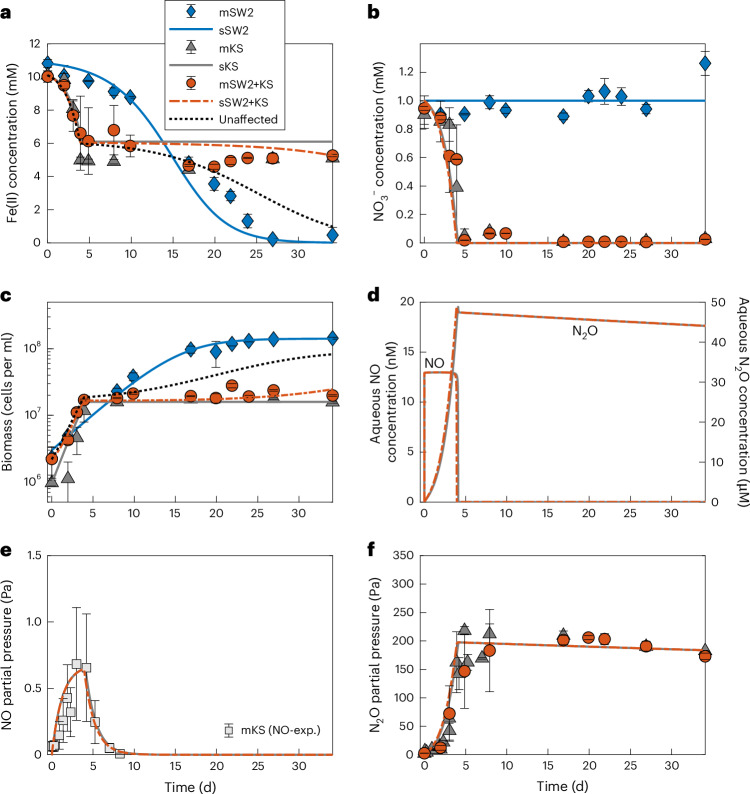
Fe(II) oxidation and nitrate reduction in the KS culture alone, *R. ferrooxidans* SW2 alone and in a mixed culture containing both KS and *R. ferrooxidans* SW2. Aqueous- and gas-phase time-series measured (m) and simulated (s) concentration data for the phototrophic oxidation of Fe(II) by *R. ferrooxidans* SW2, the nitrate-dependent oxidation of Fe(II) by KS and in the incubation containing a mixture of KS and *R. ferrooxidans* SW2. **a**, Fe(II) oxidation. **b**, NO_3_^−^ reduction. **c**, Total cell number measured via flow cytometry. **d**, Predicted aqueous concentrations of NO and N_2_O. **e**, Predicted versus measured NO partial pressure in culture KS performed during a parallel incubation (measured shown by square symbols). **f**, Predicted and measured N_2_O partial pressures. Gaseous reactive intermediates NO and N_2_O were present only in the KS and mixed KS–*R. ferrooxidans* SW2 incubations. In **a** and **c**, the dashed black lines illustrate, respectively, the expected Fe(II) and biomass growth in the absence of NO toxicity (unaffected). Data are presented as the mean ± s.d. of biological triplicates.

**Extended Data Fig. 1:**
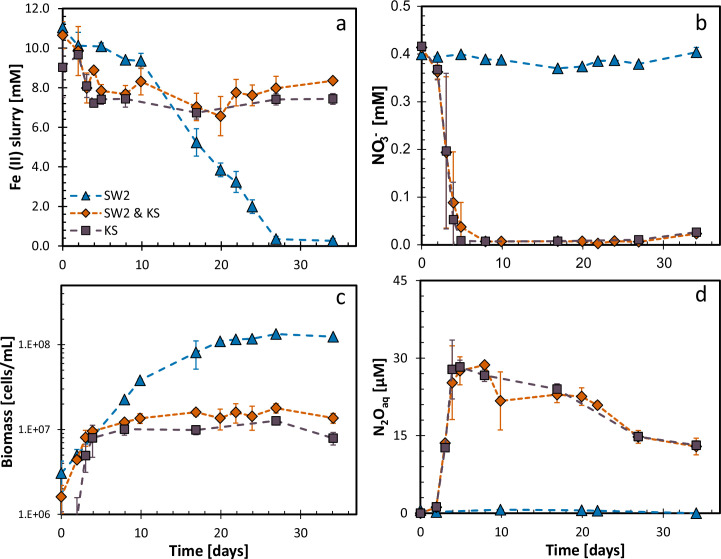
Inhibitory effect still observed at low nitrate availability. Competition dynamics between phototrophic and nitrate-reducing Fe(II)-oxidizers (*Rhodobacter ferroxidans* strain SW2 and enrichment culture KS) with limited nitrate (0.4 mM), showing that the mixed culture behaves similarly to the nitrate-reducing culture with regards to all parameters, and Fe(II) oxidation in the mixed culture is incomplete. Data presented as mean +/− standard deviation of biological triplicates. **a**, Fe(II) oxidation. **b**, NO_3_^-^ reduction, **c**, Biomass. **d**, N_2_O_aq_.

To observe potential competitive interactions we co-cultured model strains of nitrate-reducing and phototrophic Fe(II)-oxidizing bacteria and compared the rates of cell growth, Fe(II) oxidation, NO_3_^−^ reduction and N_2_O formation in the mixed culture with those grown alone. The model Fe(II) oxidizer used was the enrichment culture KS (a mixed culture that consists of a dominant Fe(II) oxidizer of the family Gallionellaceae, with a flanking community of heterotrophs including *Bradyrhizobium*, *Rhodanobacter*, *Nocardioides* and *Thiobacillus*), whereas the model photoferrotroph was the purple non-sulfur bacterium *Rhodobacter ferrooxidans* strain SW2. In all cases, CO_2_ for autotrophic growth was provided in excess for both organisms. When the photoferrotroph *R. ferrooxidans* SW2 was incubated alone with 1 mM NO_3_^−^ and 10 mM Fe(II) in the presence of light, Fe(II) oxidation was complete after 28 days and NO_3_^−^ was not consumed (Fig. 1a,b). The KS culture incubated under the same conditions reduced all available NO_3_^−^ (1 mM) over approximately four days and oxidized 5 mM of the Fe(II) (Fig. 1a,b; equation (2)). When the KS culture and photoferrotroph SW2 were incubated together, NO_3_^−^ was completely reduced but Fe(II) oxidation stopped after the consumption of approximately 4–5 mM of the Fe(II) (Fig. 1a,b). The remaining Fe(II) (5 mM) was not consumed, suggesting inhibition of the photoferrotroph in the presence of the NO_3_^−^ reducer KS. The inhibition of *R. ferrooxidans* SW2 was reflected in the cell numbers (Fig. 1c). A maximum of 10^8^ cells per ml was measured when *R. ferrooxidans* SW2 was incubated alone. Conversely, when incubated in the presence of KS, the total cell count was one order of magnitude lower. The patterns of NO_3_^−^ reduction, cell growth and Fe(II) oxidation were almost identical in the mixed culture and when KS was incubated alone, with similar trends also observed at lower NO_3_^−^ concentrations (Extended Data Fig. 1). Indeed, the KS and mixed KS–*R. ferrooxidans* SW2 incubations were so similar that Mössbauer spectra of the minerals formed are indistinguishable (Supplementary Fig. 1). We interpret these data as suggesting that the photoferrotroph did not grow in the mixed culture and that cell lysis was probably enhanced by the presence of KS.

**Fig. 2:**
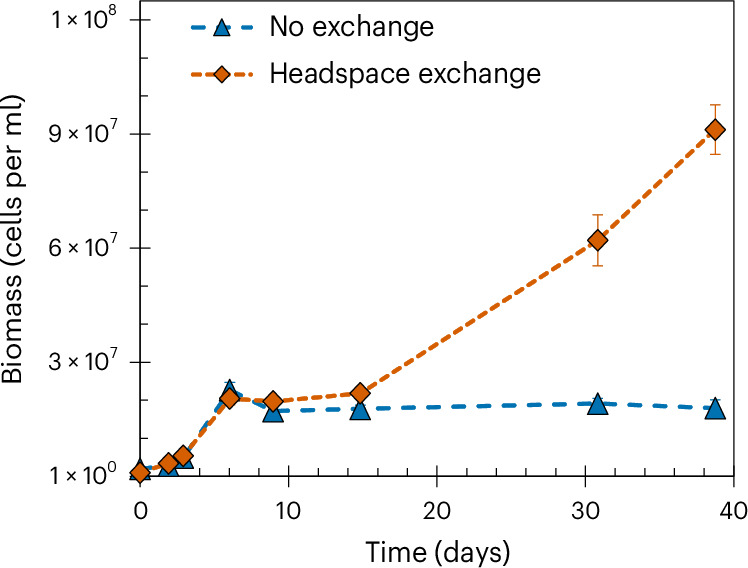
Cell numbers measured using flow cytometry in incubations that contain both KS and *R. ferrooxidans* SW2. In a KS and *R. ferrooxidans* SW2 mixed incubation, exchange of the headspace after each sampling point results in alleviation of the inhibition of phototrophic Fe(II)-oxidizers. Orange symbols denote data for which the headspace was exchanged with a N_2_/CO_2_ gas mixture after sampling, whereas blue symbols indicate no headspace exchange during the experiment. As the biomass in the KS culture is dictated by the NO_3_^−^ added, any cell growth above what can be explained by the KS culture can only be attributed to the growth of *R. ferrooxidans* SW2. Data are presented as the mean ± s.d. of biological triplicates. Dashed lines are to guide the eye and do not represent model fits.

The culture KS, which demonstrates faster Fe(II) oxidation than R. *ferrooxidans* SW2, will kinetically outcompete R. *ferrooxidans* SW2 when Fe(II) is limited, but in this situation, where Fe(II) is in excess of what can be oxidized by the available NO_3_^−^, another explanation is necessary. We evaluated whether inhibition required live and actively metabolizing KS cells, by comparing Fe(II) oxidation in the mixed culture when either live or dead (that is, autoclaved) KS cells were added (Supplementary Fig. 2). We also added a spent, filtered culture KS supernatant to an *R. ferrooxidans* SW2 culture to test whether the inhibitor had been introduced during inoculation (Supplementary Fig. 2). Combined, these experiments demonstrated that the KS culture needed to be alive and actively reducing NO_3_^−^ for inhibition to occur, suggesting that something produced during denitrification was responsible for the inhibition. The reactive intermediates NO_2_^−^, NO and N_2_O can all be potential toxins produced during denitrification^24^. However, by conducting an additional experiment where the headspace of the reactor was flushed after every sampling point, we confirmed that the inhibitor must be a highly volatile nitrogen intermediate (Fig. 2). Although we did not measure N_2_O or NO after this headspace exchange, the flushing of the headspace led to uninhibited growth in the mixed culture (Fig. 2). As the flushing of the headspace cannot provide any additional NO_3_^−^ (the ultimate limiting factor for the NO_3_^−^ reducers when both Fe(II) and CO_2_ are present in excess), enhanced cell numbers and further Fe(II) oxidation compared with the control where the headspace was not exchanged can only be explained by recovery of the photoferrotrophic community.

Recent isotope-labelling experiments demonstrated that KS produces N_2_O during denitrification^25^. Consistent with this, our experiments showed increasing concentrations of the denitrification intermediate N_2_O during NO_3_^−^ reduction, which plateaued after the sixth day in both the KS and KS–R. *ferrooxidans* SW2 incubation experiments (with a slight subsequent dilution due to ongoing sampling) (Fig. 1f). In a parallel incubation inoculated only with KS, and conducted under the same conditions, we observed that NO was also produced (in addition to N_2_O) shortly after the onset of the experiment and persisted in the system over several days, albeit at nanomolar aqueous concentrations, before being consumed (Fig. 1d,e). We did not observe any appreciable amount of NO_2_^−^ accumulation in any of the reactors, implying the efficient conversion of NO_2_^−^ to NO.

**Extended Data Fig. 2:**
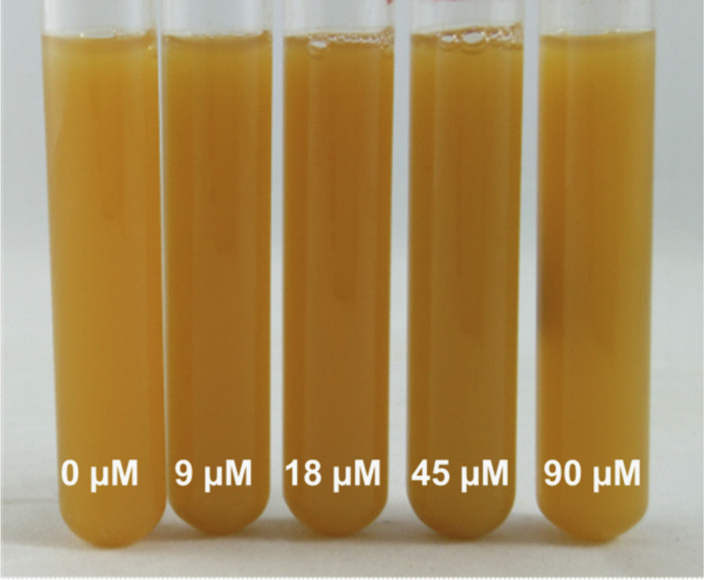
N_2_O is not toxic. Toxicity experiment with N_2_O_(aq)_ showing no toxicity and no inhibition of *Rhodobacter ferrooxidans* SW2 with N_2_O added at concentrations higher than measured in the experiment.

**Extended Data Fig. 3:**
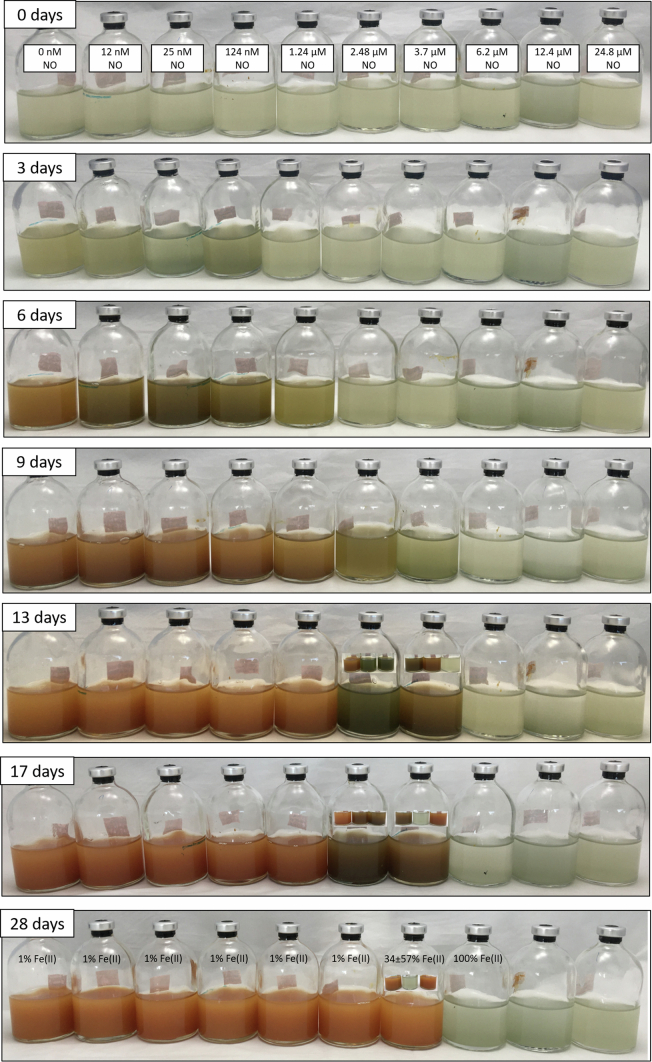
Nitric oxide shows a strong inhibitory effect. Time series of Fe(II) oxidation by R. ferrooxidans SW2 in the presence of different concentrations of nitric oxide. When 99% of Fe(II) is oxidized, bottles display characteristic orange colouration of Fe(III) minerals. Delays to iron oxidation are observed for all concentrations of NO although ultimately overcome by the end of the experiment up to 2.48 µM NO. Each bottle shown is a representative of the triplicates. Where the colour change in the triplicates varies considerably, all 3 bottles are shown as an insert.

We tested directly whether N_2_O and NO can inhibit the oxidation of Fe(II) by *R. ferrooxidans* SW2. We did not observe any inhibition by N_2_O, even at concentrations higher than those observed in Fig. 1 (up to an aqueous N_2_O concentration of 90 µM; Extended Data Fig. 2). When *R. ferrooxidans* SW2 was incubated with different concentrations of NO in the range of 12 nM to 24.8 µM, slower Fe(II) oxidation was seen at all concentrations, although all Fe(II) was eventually oxidized in concentrations up to 2.5 µM (Extended Data Fig. 3). No Fe(II) oxidation was observed at 6.2 µM. At the NO concentration of 3.7 µM, Fe(II) oxidation occurred in two of the replicates but not in the third, suggesting that the threshold may be closer to this value. We conclude from this that NO has a limiting effect on photoferrotrophy, even at very low nanomolar concentrations, although longer-term exposure may be required to induce complete inhibition at these low concentrations. This explains why recovery of the photoferrotroph cells is not observed in the mixed cultures within the time period of the experiment, whereas in the flushing experiments shown in Fig. 2 (where NO is not allowed to accumulate for any period of time) the community can recover. However, it should be noted that, even a single exposure to low nanomolar concentrations of NO can completely inhibit photoferrotrophs.

We used a numerical model to assess the feasibility of our hypothesis that the NO produced during denitrification was responsible for the observed inhibition of the photoferrotrophs (Fig. 1). When NO toxicity was considered as an inhibitor of photoferrotrophy, via enhanced cell lysis in the presence of NO, we accurately captured the measured growth and Fe(II) dynamics. In the absence of NO toxicity effects (Fig. 1a,c, indicated by the ‘unaffected’ dotted line), the model overestimated both biomass growth and the extent of Fe(II) oxidation. Our model predicts that NO toxicity leads to a rapid decay in photoferrotrophic cell numbers over the first five days of the experiment while KS is actively growing, followed by a slow rebound over 30 days or more (Supplementary Fig. 3).

## Potential ubiquity of inhibitory effect

**Fig. 3:**
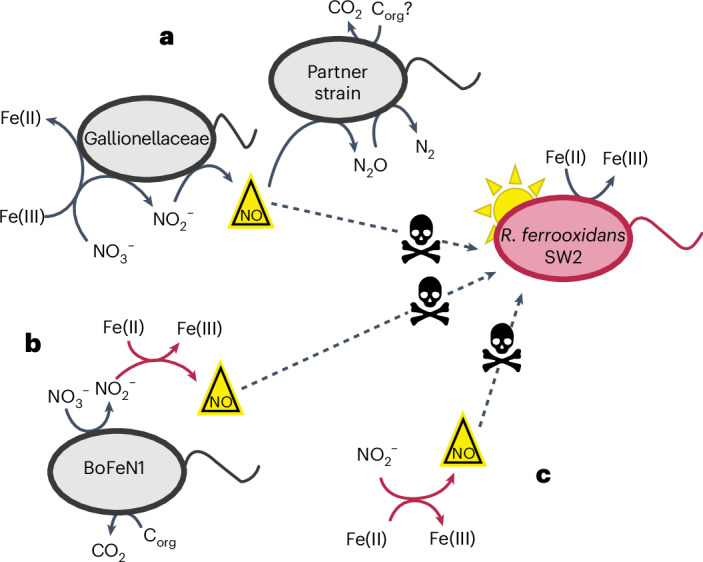
Schematic of NO production and phototroph inhibition by NO via three mechanisms of nitrate-reducing Fe(II) oxidation. **a**, The Fe(II) oxidizer of culture KS (*Gallionella*) produces NO via denitrification coupled with enzymatic Fe(II) oxidation. Some NO may be scavenged by the heterotrophic partner strains (dominated by *Bradyrhizobium*), although this is probably limited by carbon availability. Remaining NO could interact with the phototroph causing inhibition. It is also possible that some NO reacts with Fe(II) abiotically in this scenario. The measured NO concentrations reflect the balance of these competing production and consumption pathways. Importantly, our model does not simulate the individual contribution of the components of the KS culture but models the culture as a whole. C_org_, organic carbon. **b**, Fe(II) oxidation by *Acidovorax* sp. BoFeN1 produces NO_2_^−^ during heterotrophic denitrification, which oxidizes Fe(II) abiotically and produces NO. **c**, Nitrite reacts abiotically with Fe(II) and produces NO. In all cases, the NO produced inhibits photoferrotrophic activity.

**Extended Data Fig. 4:**
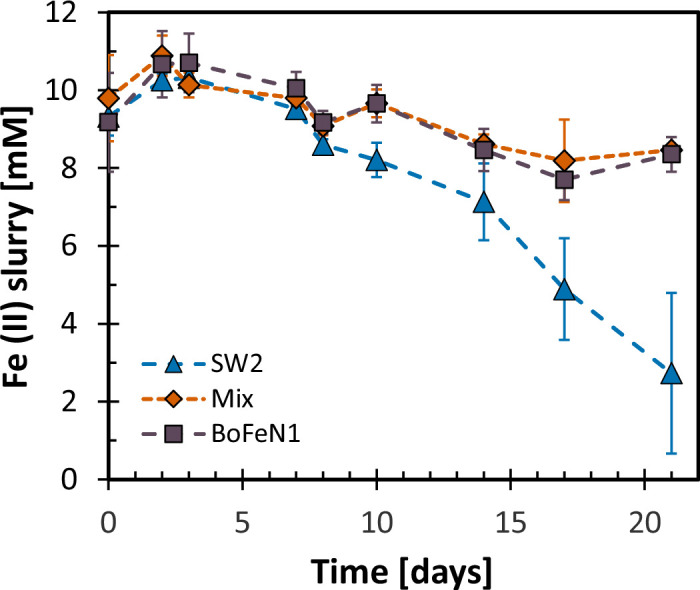
Inhibition also observed with another nitrate-reducing Fe(II)-oxidizer. Fe(II) oxidation by *R. ferrooxidans* SW2, *Acidovorax* sp. BoFeN1 and a mixed incubation containing both. Inhibition of SW2 is also observed when this alternative nitrate-reducing Fe(II)-oxidizer is used. Data presented as mean +/− standard deviation of biological triplicates.

**Extended Data Fig. 5:**
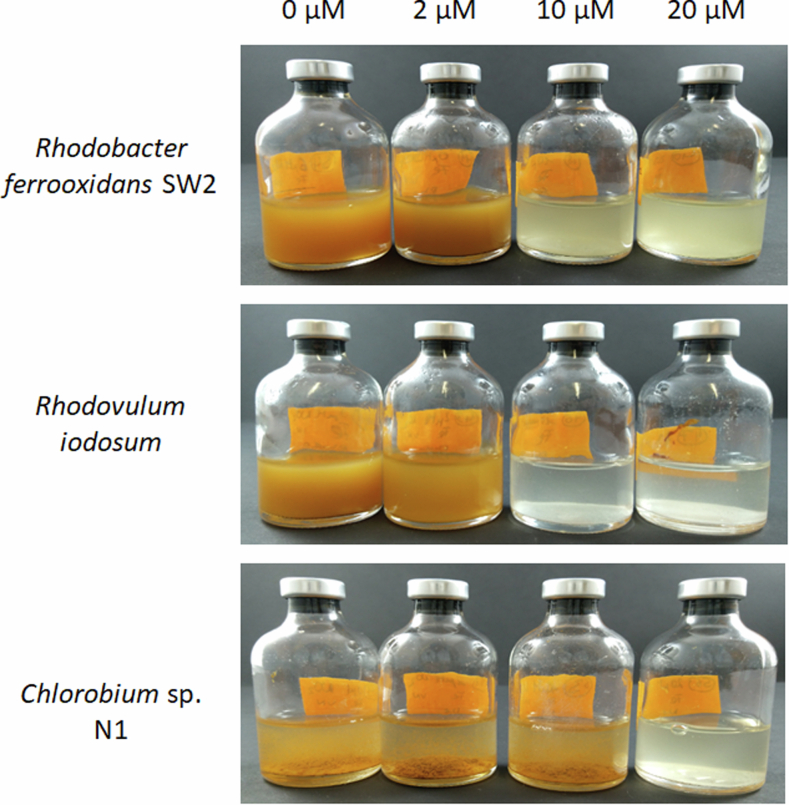
Inhibition can occur during abiotic reactions between nitrite and Fe(II). Photoferrotrophs *R. ferrooxidans* SW2 (top), *Rhodovulum iodosum* (middle) and *Chlorobium* sp. N1 (bottom) grown alone with different nitrite concentrations (listed at the top of the figure). All strains show inhibition suggesting the abiotic reaction between nitrite and Fe(II) can be inhibitory if the nitrite concentrations are high enough. 10 mM Fe(II) was added to all cultures.

If the inhibition effect observed during co-culturing with the KS–*R. ferrooxidans* SW2 mixture is caused by the production of NO as we suggest, the effect will not be unique to culture KS and will also be observed in other reactions between Fe(II) and nitrogen species. We further investigated the effect of microbially catalysed chemodenitrification using *Acidovorax* sp. BoFeN1, and entirely abiotic Fe(II) oxidation with NO_2_^−^ (Extended Data Figs. 4 and 5 and Supplementary Results 1), and show that both lead to inhibition of the photoferrotroph *R. ferrooxidans* SW2. In all cases, the inhibition can be best explained by the production of highly toxic NO as an intermediate of denitrification under ferruginous conditions (Fig. 3).

**Extended Data Fig. 6:**
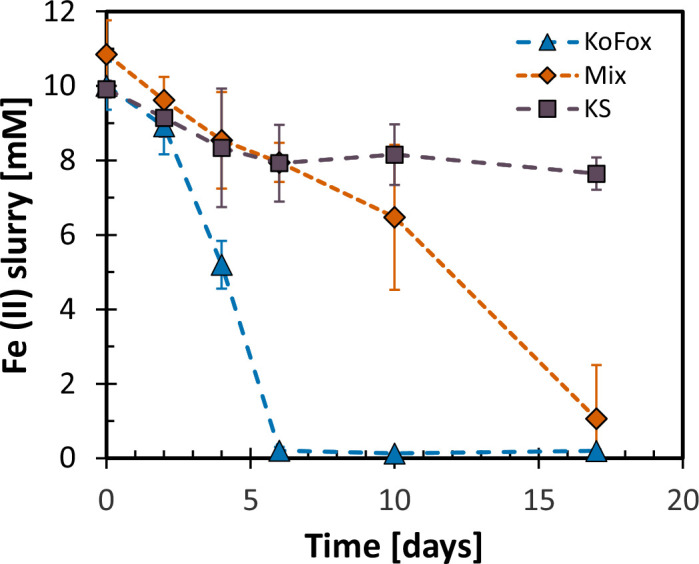
Green Sulfur Bacteria are also inhibited by culture KS. Fe(II) oxidation by culture KS, *Chlorobium ferroxidans* sp. KoFox (a freshwater GSB) and an incubation containing both. In this case 0.4 mM NO_3_^−^ was added. *Chlorobium ferrooxidans* sp. KoFox is also inhibited by KS, but eventually overcomes the inhibition. We suggest the mechanism of inhibition is the same as for *R. ferrooxidans* SW2. Data presented as mean +/− standard deviation of biological triplicates.

Furthermore, we tested whether inhibition of Fe(II) oxidation occurs when an alternative freshwater photoferrotroph, *Chlorobium ferrooxidans* strain KoFox, was incubated with culture KS. In this case, Fe(II) oxidation was delayed but not completely inhibited (Extended Data Fig. 6). We also observed that two marine photoferrotrophs (*Chlorobium* sp. N1 and *Rhodovulum iodosum*) were sensitive to abiotic chemodenitrification processes in the presence of Fe(II). *Chlorobium* sp. N1 oxidized Fe(II) in the presence of 2 and 10 µM of NO_2_^−^ (to promote NO formation via abiotic reaction with Fe(II)) but not with NO_2_^−^ at 20 µM; *R. iodosum* oxidized Fe(II) in the presence of 2 µM of NO_2_^−^, but not with 10 or 20 µM (Extended Data Fig. 5). The Fe(II) oxidation mechanism in this case is of the type depicted in Fig. 3c. The sensitivity of these marine strains highlights that we also expect to observe a similar effect in the marine realm.

All of the strains tested have some genetic capability for tolerating NO. The photoferrotrophs *C. ferrooxidans* KoFox, *Chlorobium* sp. N1 and *R. iodosum* all possess the *norV* gene, encoding a flavorubredoxin which reduces NO for detoxification purposes^26^. *R. ferrooxidans* SW2 contains the *norB* gene, encoding the canonical NO reductase in the denitrification pathway. However, it is more likely that this canonical NO reductase has a detoxification role in strain SW2, which is incapable of denitrification (as demonstrated in Fig. 1). This suggests either that the possession of NO reduction genes, regardless of type, does not accurately predict the ability of a strain to tolerate NO or that the concentrations of NO produced in our experiments are outside the range in which NO can be efficiently detoxified.

**Extended Data Fig. 7:**
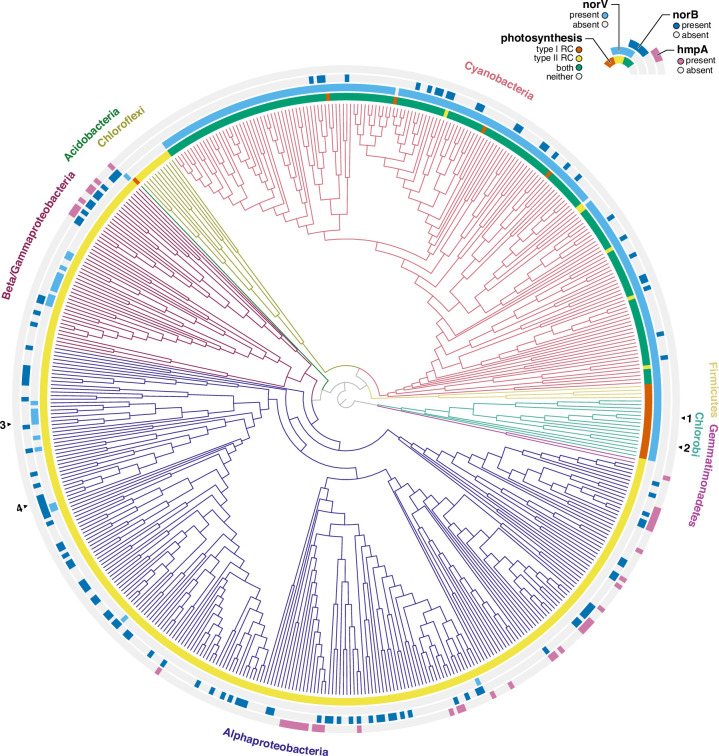
NO defense genes are common across photosynthetic bacteria. Distribution of nitric oxide reduction genes in genomes of phototrophic bacteria. Anoxygenic phototrophs were identified by possession of genes encoding for Type I (pshA) or Type II (pufL) reaction centers. Cyanobacteria are identified by the presence of genes for both types of reaction center, photosystem I (psaB) and photosystem II (psbA). The presence of norV, norB and hmpA are indicated by light blue, dark blue, or pink annotations, respectively. NorV is ubiquitous in the Chlorobi and Cyanobacteria but is less common in anoxygenic phototrophs with Type II reaction centers. Numbers denote positions of the 4 photoferrotrophs cultured in this study: (1) Chlorobium ferrooxidans KoFox, (2) Chlorobium sp. N1, (3) Rhodovulum iodosum, and (4) Rhodobacter ferrooxidans SW2. Branches were collapsed based on a 16S % identity threshold of 97%. Tips representing the four strains cultured in this study were kept as individual tips, regardless of their identity score.

Comparative genomic analysis of NO detoxification abilities across phototrophs (Supplementary Results 2) suggests that there are differences in genetic strategies for NO detoxification amongst different groups of phototrophs, and emphasizes that our cultured photoferrotrophs (Extended Data Fig. 7) are broadly representative of their respective groups. This implies that the inhibition we observe in culture is probably not limited to our tested strains but that all phototrophs may be vulnerable despite having some genetic ability to detoxify NO. In addition, the ubiquity of *norV* amongst the genomes of extant Cyanobacteria and Chlorobi species suggests that this gene may have evolved early in the history of these groups (Extended Data Fig. 7 and Supplementary Fig. 4).

## Inhibition of photoferrotrophy in ancient oceans

**Fig. 4:**
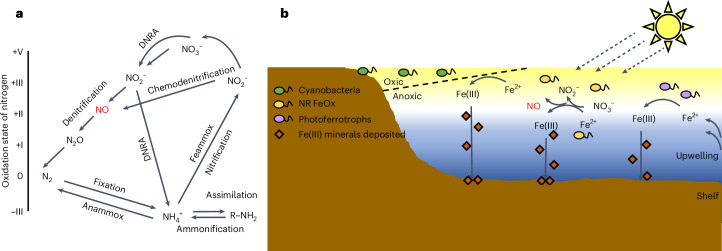
Microbial mechanisms of NO production and the proposed impact of nitrate-reducing Fe(II) oxidation on BIF formation. **a**,**b**, Schematics demonstrating the variety of pathways in the nitrogen cycle that can lead to production of toxic NO (**a**) and the potential marginalization of photoferrotrophs by nitrate-reducing Fe(II) oxidizers in an early stratified continental shelf with cyanobacteria beginning to colonize benthic mats, produce O_2_ and enhance the availability of NO_3_^−^ (**b**). The vertical scale of the shelf is exaggerated. DNRA, dissimilatory nitrate reduction to ammonium; anammox, anaerobic ammonium oxidation; Feammox, anaerobic ammonium oxidation coupled with Fe(III) reduction; NR FeOx, nitrate-reducing Fe(II)-oxidizing microorganisms.

Measured NO concentrations in modern ferruginous systems are highly variable but can reach up to 500 nM, for example, in anoxic sediments^27,28^. In these settings NO may be produced as a by-product of microbial denitrification or chemodenitrification but is also an intermediate of nitrification and ammonium oxidation^29^ (Fig. 4a). We demonstrated that NO concentrations during KS-mediated denitrification can accumulate up to 13 nM (Fig. 1), which is about 30-fold lower than the highest concentrations measured in some modern anoxic settings, and thus NO sensitivity can be expected at environmentally relevant concentrations (for an extended discussion see Supplementary Discussion 1).

Ferruginous environments were much more common in the Earth’s past, and thus the effects we report here were probably more important earlier in Earth’s history. The complete biological cycle of nitrogen fixation, nitrification and denitrification (including chemodenitrification) had probably evolved by the late Archaean or early Proterozoic^8,9,30,31^, although it is thought that up to 10^13^ grams of NO per year could have been produced by atmospheric photochemical reactions as far back as the Hadean^32^. The existence of microbially driven nitrate-reducing Fe(II) oxidation—which linked the iron and nitrogen biogeochemical cycles—is one possibility that has been evoked to explain the inverse co-variations between δ^15^N and δ^13^C_carbonate_ isotopes recorded in the Brockman Iron Formation of the early Palaeoproterozoic in Western Australia^6^. In an alternative scenario, these authors propose that iron-driven chemodenitrification in a redox-stratified water column could drive the observed signatures. Furthermore, although inferred from mineralogical assemblages rather than direct from isotopic measures, NO_3_^−^ reduction through organic carbon oxidation is thought to best explain the observed mineralogy and geochemistry in the ~2.9 Ga Nconga Iron Formation (Mozaan Group, South Africa), which would push the potential for nitrate-reducing Fe(II) oxidation (NRFO) back even further in time^7^. Our results demonstrate that nitrate-reducing Fe(II) oxidation could have the potential to inhibit the activity of photoferrotrophs in systems similar to those that produced the Brockman Iron Formation. Considering that photoferrotrophs are thought to have contributed to BIF formation as early as 3.77 Ga (refs. ^33,34^), and that NO_3_^−^ was likely to be at least locally available as early as 2.7 Ga (ref. ^9^), there is a potentially long time frame from then until the onset of the GOE (2.45 Ga) within which photoferrotrophs could have encountered microbial or abiotic nitrate-reducing Fe(II) oxidation in the photic zone. On the basis of the ubiquity of photoferrotrophs for which NO is toxic, our findings imply, at the very least, that nitrate-reducing Fe(II) oxidation could have imposed a selection pressure on strains with detoxifying capabilities or provided an impetus to evolve NO detoxifying traits. At worst, nitrate-reducing Fe(II) oxidation could have created photoferrotroph exclusion zones in regions with elevated NO_3_^−^ availability.

The potential marginalization of photoferrotrophs by nitrate-reducing Fe(II) oxidizers represents a previously unknown control on mechanisms of BIF deposition. As the marine photic zone became progressively oxygenated before the GOE, one of the immediate outcomes would have been the production of NO_3_^−^ in marine settings with high primary productivity. This would have been followed by the proliferation of either chemodenitrification or enzymatic denitrification, with the two processes influencing photoferrotrophs via metabolic competition for Fe(II) as the electron donor, the production of NO as a toxin or a combination of the two. Under ferruginous conditions, dissimilatory nitrate reduction to ammonium would also probably have played a role that could counteract the toxicity effect through by-passing the intermediate steps in canonical denitrification. However, subsequent anaerobic oxidation of this ammonium (via Feammox) would produce NO_2_^−^ as an intermediate, which could then interact with Fe(II) via chemodenitrification and produce NO (Fig. 4a).

In terms of BIF deposition, we envisage an Archaean ocean where photoferrotrophs were the primary biological driver of Fe(II) oxidation in the photic zone. However, as NO_3_^−^ became more abundant and denitrification intensified, the photoferrotrophs would have been pushed further offshore, away from areas of peak primary productivity where cyanobacteria grew as mats^35,36^, and where O_2_ and oxidized nitrogen species were accumulating. Although the photoferrotrophs would still have had first access to upwelling Fe(II)^4,37^, they would have found themselves limited by other trace elements sourced from continental weathering. We hypothesize that nitrate-reducing Fe(II) oxidation could have plausibly compensated for much of the BIF deposition after photoferrotrophs became inhibited (Supplementary Discussion 2), although aerial expansion of cyanobacteria and oxygenation of the deep ocean would ultimately have resulted in the cessation of BIF.

Finally, as O_2_ increasingly diffused away from coastal environments, the oxic zone would have eventually intersected with the photic zone. As photoferrotrophs are obligate anaerobes, this O_2_ would have completely limited their ability to survive in the open oceans. Consequently, upwelling Fe(II) would no longer have been oxidized via photoferrotrophy. At this stage, Fe(II) oxidation would instead have been driven by microaerophilic chemolithoautotrophs (for example, *Gallionella*), nitrate-reducing Fe(II) oxidizers or abiotic Fe(II) oxidation with O_2_ or reactive nitrogen species.

In summary, our work demonstrates that NO produced by denitrifying bacteria can influence the survival of photoferrotrophs, and highlights that this toxicity would be enhanced under ferruginous conditions. The levels of NO required to inhibit photoferrotrophy are low and within the range observed in modern ferruginous environments, suggesting that NO stress could have played an important role in shaping the biogeochemistry and microbial community in both modern and ancient ferruginous habitats with an active nitrogen cycle. It is often thought that the main challenge for photoferrotrophs arose when O_2_ became more widespread. However, the introduction of reactive nitrogen species, such as NO_2_^−^ and NO, into a ferruginous world would also have made photoferrotrophy difficult. Local enrichment of NO may have influenced biogeochemical cycling and, via inhibition of mineral precipitating phototrophs, fundamentally altered the mechanisms of BIF deposition, one of the main records of early ocean biogeochemistry itself.

## Methods

### Model strains

All experiments described in the main text were conducted using the autotrophic nitrate-reducing Fe(II)-oxidizing enrichment culture KS^17^ and the phototrophic Fe(II) oxidizer *R. ferrooxidans* SW2^38^. Culture KS was enriched from a ditch in Bremen, Germany, and *R. ferrooxidans* SW2 was isolated from freshwater sediments in Hannover, Germany. Both cultures were maintained in continuous culture in the culture collection of A.K. The media recipes for other strains used can be found in Supplementary Table 2.

### Cultivation

For cultivation of KS and *R. ferrooxidans* SW2, 22 mM bicarbonate-buffered mineral medium was used for all set-ups and contained KH_2_PO_4_ (0.6 g l^−1^), NH_4_Cl (0.3 g l^−1^), MgSO_4_·7H_2_O (0.025 g l^−1^), MgCl_2_·6H_2_O (0.4 g l^−1^) and CaCl_2_·2H_2_O (0.1 g l^−1^). After autoclaving in a Widdel flask the medium was cooled to room temperature under an N_2_/CO_2_ atmosphere (90:10) and buffered with anoxic 22 mM bicarbonate buffer. The CO_2_ concentrations in the final medium were in excess of the requirements for both the KS culture and the growth of *R. ferrooxidans* SW2. Aliquots (1 ml l^−1^) of sterile filtered seven-vitamin solution, trace element solution^39^ and selenite-tungstate solution^40^ were added and the pH was adjusted to 7 with 0.5 M NaHCO_3_ or 1 M HCl. The medium was stored at 5 °C. Before each experiment, the medium was aliquoted into sterile glass serum vials with a 50% headspace consisting of N_2_/CO_2_ gas (90:10) and amended with FeCl_2_·4H_2_O and NaNO_3_ as required. The pre-culture was always grown under Fe(II)-oxidizing conditions. In all cases, a 1% inoculum of both the KS and *R. ferrooxidans* SW2 pre-cultures was used, which equates to a starting cell concentration for KS of 1 × 10^6^ ± 1 × 10^5^ cells per ml and for *R. ferrooxidans* SW2 of 2 × 10^6^ ± 1 × 10^6^ cells per ml (although in a supplementary experiment we observed that inhibition of *R. ferrooxidans* SW2 was independent of the starting ratio of SW2:KS cells; Supplementary Fig. 5). Serum vials were placed in a light incubator with 24 h light that reached 23 ± 3 µmol m^−2^ s^−1^ (2,700 K) at 25 °C. The vial position was randomized after each sampling point to avoid enhancing any effects caused by incubator position. Medium recipes for the other strains tested (*Acidovorax* sp. BoFeN1, *Chlorobium* sp. N1 and *R. iodosum*) are shown in Supplementary Table 2.

### Competition between KS and *R. ferrooxidans* SW2

To evaluate potential competitive effects between KS and *R. ferrooxidans* SW2, a sample of the medium (50 ml) was amended with 10 mM FeCl_2_ and 0.4 mM (scenario A) or 1 mM NaNO_3_ (scenario B). These concentrations are high compared with what is expected in modern or ancient environments but are a practical necessity to monitor the competitive dynamics. Three different conditions were tested: KS alone, *R. ferrooxidans* SW2 alone and KS and *R. ferrooxidans* SW2 grown together in the same vial. At every sampling time point, samples for gas analyses were collected under sterile conditions at the laboratory bench before samples for Fe, NO_2_^−^, NO_3_^−^ and cell counts were taken using an anoxic glovebox under a 100% N_2_ atmosphere.

### Further control experiments

The potential for inhibition by the presence of KS biomass or a component from the supernatant was evaluated (Supplementary Fig. 2). For this, we repeated the experiment but inoculated additional triplicates with cells from an autoclaved pre-culture (121 °C, 20 mins) or with an equal volume of pre-culture supernatant that had been passed through a 0.22 µm filter. Samples for Fe, NO_2_^−^, NO_3_^−^, N_2_O and cell number were collected at every time point as described in the previous section.

### Toxicity tests

To determine whether potentially toxic gaseous products were causing inhibition of *R. ferrooxidans* SW2 when grown in combination with KS, we prepared six vials as described in the experiment above with 10 mM FeCl_2_ and 1 mM NaNO_3_. Three vials were flushed with an N_2_/CO_2_ gas mixture for 5 min after every sampling time point or every other day. The remaining three vials did not have the headspace replenished at any point (Fig. 2).

The potential for N_2_O toxicity in *R. ferrooxidans* SW2 was tested by inoculation into different concentrations of N_2_O with 10 mM FeCl_2_ as the electron donor. These tests were performed in 15 ml Hungate tubes with medium (9 ml) and inoculum (1 ml) in triplicates. Fe(II) oxidation was monitored visually, with positive growth demonstrated by a colour change from grey to orange. The concentration range of N_2_O(aq) was from 0 to 90 µM (0, 9, 18, 45 and 90 µM) (Extended Data Fig. 2).

The potential for NO toxicity in *R. ferrooxidans* SW2 was tested by inoculation into different concentrations of NO in triplicate with 10 mM FeCl_2_ as the electron donor. Microbial Fe(II) oxidation was monitored over time via a colour change from grey to orange, with the extent of Fe(II) oxidation measured at the end of the experiment (time-course sampling was not conducted so as to avoid potential dilution of the NO added) (Extended Data Fig. 3).

Different concentrations of NO_2_^−^ (0, 2, 10 and 20 µM) were tested in combination with 10 mM FeCl_2_, in duplicates, to determine the potential for NO_2_^−^ toxicity. Microbial Fe(II) oxidation was indicated by a colour change from grey to orange. NO_2_^−^ toxicity was tested for three phototrophic Fe(II) oxidizers in total: *Rhodobacter ferrooxidans* SW2, *Rhodovulum iodosum* and *Chlorobium* sp. strain N1 (Extended Data Fig. 5; see Supplementary Information for culture conditions for additional strains). The toxicity of NO_2_^−^ in the absence of Fe(II) was determined by incubating *R. ferrooxidans* SW2 with acetate as an alternative growth substrate (Supplementary Fig. 6). Growth was monitored via the optical density at 660 nm in the presence of 0, 0.5 or 2 mM NaNO_2_.

### Iron quantification

Iron (Fe(II) and Fe(III)) was quantified spectrophotometrically using the ferrozine assay of Stookey^41^. Owing to the potential presence of NO_2_^−^, the protocol was modified and 1 M HCl was used together with 40 mM sulfamic acid^18,42^ to stabilize iron from abiotic reactions with nitrogen species. The Fe(III) data did not inform the results (although they did confirm that Fe(II) removal was as a result of oxidation and not, for example, sorption to the glass wall) and thus are not shown in the results. During sampling, the sample (0.1 ml) was added to a 0.9 ml aliquot of 40 mM sulfamic acid in 1 M HCl. Samples were stored at 5 °C until quantification. The ferrozine–Fe(II) complex was quantified at 562 nm using a microtitre plate reader (Multiskan GO, Thermo Fisher Scientific). Ferrozine measurements were conducted in triplicate.

### Cell quantification

Cells were counted using a flow cytometer equipped with a 488 nm laser as the excitation source (Attune Nxt flow cytometer, Thermo Fisher Scientific). Samples for cell counts were directly processed after sampling. Sterile filtered oxalate solution (600 µl) was added to the sample (200 µl) and incubated for between 30 s and 1 min. A 1,200 µl aliquot of 10 mM sterile filtered bicarbonate buffer was added, and the mixture was centrifuged at 21,130*g* for 10 min. A portion of the supernatant (1,800 µl) was discarded and 10 mM sterile filtered bicarbonate buffer (600 µl) was added. BacLight Green stain (Thermo Fisher Scientific, 1 µl stain per ml of sample) was added and a 200 µl aliquot of the sample was distributed in triplicate in 96-well plates. The plate was incubated for 15 min in the dark before measurements were taken. Cells were distinguished from noise or debris on the basis of their properties in the side scatter and BL1 channel (with emission filter 530/30 nm). This method measures the total cell numbers and does not distinguish between different species.

### N_2_O quantification

Samples were extracted from the headspace using a Hamilton syringe after each bottle was shaken and transferred into vials previously flushed with N_2_. The vials were stored at room temperature until further analysis. The analysis was performed using a gas chromatograph with a pulsed discharge detector (PDD). The temperature programme for the columns was: 10 min at 35 °C, heated at 50 °C min^−1^ until 120 °C and held at 120 °C for 1 min. This was repeated with heating at 50 °C min^−1^ until 150 °C and held at this temperature for 5 min. The valve furnace was set to 40 °C. The carrier gas flow was 5 ml min^−1^ and the run time was 18.3 min. The back PDD used a Mol Sieve 5A column (30 m × 0.53 mm (length × inner diameter, respectively)) and the front PDD used a TG BondQ+ column (30 m × 0.25 mm). The injection volume was 2 ml.

### NO_2_^−^ and NO_3_^−^ quantification

Samples for NO_2_^−^ and NO_3_^−^ were taken using a glovebox, centrifuged at 21,130*g* for 5 min and then stored under anoxic conditions at 5 °C until measurement. Concentrations were quantified colorimetrically using a continuous-flow analyser (Seal Analytical). For details, see ref. ^43^.

### NO monitoring

To determine whether NO accumulated during growth of the KS culture we conducted a parallel incubation with only KS inoculated in the reactor but under the same conditions as in the original experiment. This was necessary as the samples must be analysed as soon as possible after sampling and thus had to be conducted in the vicinity of the instrument, located at the University of Arizona where the culturing of anoxygenic phototrophs was not possible at the time. For this incubation, cells pre-grown with Fe(II) as an electron donor were transferred (0.5% inoculum) into reactors containing fresh medium with 10 mM FeCl_2_, 1 mM NaNO_3_ and a N_2_/CO_2_ (90:10) headspace. The cultures were incubated in the dark at 25 °C, and NO and N_2_O evolution was followed over time. NO was quantified in the microcosm headspace using a chemiluminescence-based analyser (LMA-3D NO_2_ analyser, Unisearch Associates). Headspace gas (50 µl) was sampled by replacement under sterile conditions using a CO_2_-N_2_-flushed gas-tight syringe and injected into the analyser. The injection port was customized to fit the injection volume and consisted of a T-junction with an air filter at one end and a septum at the other end. An internal pump generated a consistent airflow. Our method generally followed a previous protocol^44^ and included adjustments based on our experimental set-up. In short, NO was oxidized to NO_2_ using a CrO_3_ catalyst. The NO_2_ passed across a fabric wick saturated with a luminol solution (luminol was obtained from Drummond Technology). Readings were corrected for background NO_2_ every 15 min. The shell airflow rate was kept at 500 ml min^−1^ and the span potentiometer was set to 8. Measurements were calibrated using a 0.1 ppm NO (in N_2_) standard (<0.0005 ppm NO_2_, Scott-Marin) over a range of 50–10,000 ppb.

### Mössbauer spectroscopy

Samples for ^57^Fe Mössbauer spectroscopy were prepared inside an anoxic (100% N_2_ atmosphere) glovebox by passing the sample through a 0.45 µm filter and sealing the filter paper between two pieces of airtight Kapton tape. The samples were stored in airtight (100% N_2_ atmosphere) bottles at −20 °C until analysis. The bottles were opened just before loading the samples inside a closed-cycle exchange gas cryostat (Janis Cryogenics) under a backflow of helium. Spectra were collected at 77 K with a constant acceleration drive system (WissEL) in transmission geometry with a ^57^Co/Rh source and calibrated against a 7-µm-thick α-^57^Fe foil measured at room temperature. All spectra were fitted applying a Voigt-based fitting routine^45^ using Recoil software (University of Ottawa)^45^. The half-width at half-maximum (HWHM) was fixed at 0.13 mm s^−1^ for all samples.

### Reaction model

Incubation reactors for all experimental treatments, photoferrotrophy by *R. ferrooxidans* SW2, photoferrotrophy and nitrate-reducing Fe(II) oxidation (*R. ferrooxidans* SW2 plus KS) and KS-mediated nitrate-reducing Fe(II) oxidation were all simulated as well-mixed batch reactors. The model variants simulate microbially mediated reactions considering Monod kinetics that explicitly account for biomass.

The growth rate of *R. ferrooxidans* SW2 (*r*_SW2_) during photoferrotrophy, in the presence of a continuous light source, was modelled via a single Monod rate expression (equation (3)):

3 $${r}_{{{\mathrm{SW}}}2}={\mu }_{\max }^{{{\mathrm{ph}}}}\left(\frac{{C}_{{{\mathrm{Fe}}}\left({{\mathrm{II}}}\right)}}{{C}_{{{\mathrm{Fe}}}\left({{\mathrm{II}}}\right)}+{K}_{{{\mathrm{Fe}}}\left({{\mathrm{II}}}\right)}^{{{\mathrm{ph}}}}}\right){X}_{{{\mathrm{SW}}}2}$$

where ${\mu }_{\max }^{{\mathrm{ph}}}$ (per day) is the maximum specific growth rate constant for photoferrotrophy, *C*_Fe(II)_ (mM) is the concentration of aqueous Fe(II), ${K}_{{{\mathrm{Fe}}}\left({{\mathrm{II}}}\right)}^{{\mathrm{ph}}}$ (mM) is the half-saturation constant for photoferrotrophy and *X*_SW2_ (cells per litre) is the biomass density of suspended *R. ferrooxidans* SW2. The equation for *r*_SW2_ assumes that light is non-limiting. The corresponding changes in biomass density and Fe(II) concentration with respect to time are given by:

4 $$\frac{{\mathrm{d}}{X}_{{{\mathrm{SW}}}2}}{{{\mathrm{d}}t}}={r}_{{{\mathrm{SW}}}2}$$

5 $$\frac{{\mathrm{d}}{C}_{{{\mathrm{Fe}}}({{\mathrm{II}}})}}{{{\mathrm{d}}t}}=-\frac{{r}_{{{\mathrm{SW}}}2}}{{Y}_{{{\mathrm{SW}}}2}}$$

where *Y*_SW2_ (cells per mmol of Fe(II)) is the growth yield of *R. ferrooxidans* SW2 on Fe(II).

Nitrate-reducing Fe(II) oxidation by KS was modelled considering two denitrification steps (${{\rm{NO}}}_{3}^{-}\mathop{\longrightarrow }\limits^{1}{\rm{NO}}\mathop{\longrightarrow }\limits^{2}{{\rm{N}}}_{2}{\rm{O}}$), where the dependence of the electron acceptor (N species) and electron donor (Fe(II)) was accounted for via dual-Monod kinetics. We chose to constrain our model to two reaction steps on the basis of the results described previously^25^, which suggest that KS may not be able to reduce N_2_O to N_2_, and the fact that no NO_2_^−^ was detected in our experiments, suggesting fast kinetics for the step from NO_3_^−^ to NO_2_^−^. Although there is abundant discussion in the literature regarding which strain in the enrichment culture is responsible for each denitrification step, and the extent to which each step is enzymatically coupled to Fe(II) oxidation^16,43,46^, we adopted the simplest scenario in the simulations, which does not distinguish between community members and assumes that all denitrification steps are enzymatically coupled to Fe(II) oxidation. The true scenario may be more complex; however, our model formulation successfully captured the dynamics of the culture well and accurately predicted the timing and magnitude of the formation of reactive intermediates.

The growth rate of KS, ${r}_{{{\mathrm{KS}}}}^{i}$, during each denitrification step is given by the following generalized expression:

6 $${r}_{{{\mathrm{KS}}}}^{\,i}={\mu }_{\max }^{i}\left(\frac{{C}_{{{\mathrm{Fe}}}\left({{\mathrm{II}}}\right)}}{{C}_{{{\mathrm{Fe}}}\left({{\mathrm{II}}}\right)}+{K}_{{{\mathrm{Fe}}}\left({{\mathrm{II}}}\right)}^{{{\mathrm{NDFO}}}}}\right)\left(\frac{{C}_{{{\mathrm{N}}}_{i}}}{{C}_{{{\mathrm{N}}}_{i}}+{K}_{{{\mathrm{N}}}_{i}}}\right){X}_{{{\mathrm{KS}}}}$$

where ${\mu }_{\max }^{i}$ (per day) is the maximum specific growth rate constant for the reduction of nitrogen species, *i*, coupled to Fe(II) oxidation, ${K}_{{{\mathrm{Fe}}}\left({{\mathrm{II}}}\right)}^{{{\mathrm{NDFO}}}}$ (mM) is the half-saturation constant for NDFO, ${K}_{{{\mathrm{N}}}_{i}}$ (mM) is the half-saturation constant for each *i*th electron acceptor (either NO_3_⁻ or NO) in the denitrification chain and *X*_KS_ (cells per litre) is the biomass density of suspended KS. Each step in the denitrification chain was modelled as a microbially mediated step, assumed to be carried out by a facet of the KS culture. Abiotic reaction steps were not accounted for in our model formulation. Kinetic mass transfer between the aqueous and gaseous phases (headspace and liquid) was simulated via linear-driving-force film diffusion, assuming Henry’s law partitioning of NO, N_2_O and N_2_.

7 $${r}_{{{\mathrm{tr}}}}^{\,i}={k}_{{{\mathrm{tr}}}}\left({C}_{{{\mathrm{N}}}_{i}}-\frac{{p}_{{{\mathrm{N}}}_{i}}}{{RT}{H}_{i}}\right)$$

In equation (7), ${r}_{\rm{tr}}^{i}$ (mM per day), is the mass transfer rate between headspace and liquid of the *i*th volatile nitrogen compound, *k*_tr_ (per day) is the first-order mass-transfer rate coefficient, ${C}_{{{\mathrm{N}}}_{i}}$ (mM) is the aqueous-phase concentration, ${p}_{{{\mathrm{N}}}_{i}}$ (Pa) is the partial pressure and *H*_*i*_ is the Henry’s law constant of the *i*th volatile nitrogen compound, respectively; *R* (J mol^−1^ K^−^^1^) is the ideal gas constant and *T* is the absolute temperature (298.15 K). (Note that headspace dilution due to sampling was also considered.) The aqueous concentration changes for Fe(II), the nitrogen species and KS are given by:

8 $$\frac{{\mathrm{d}}{C}_{{{\mathrm{Fe}}}\left({{\mathrm{II}}}\right)}}{{{\mathrm{d}}t}}=-\mathop{\sum }\limits_{i=1}^{n}\frac{{r}_{{{\mathrm{KS}}}}^{\,i}}{{Y}_{{{\mathrm{KS}}},i}}$$

9 $$\frac{{\mathrm{d}}{C}_{{{\mathrm{NO}}}_3}}{{{\mathrm{d}}t}}=-\frac{1}{3}\frac{{r}_{{{\mathrm{KS}}}}^{{{\mathrm{NO}}}_3}}{{Y}_{{{\mathrm{KS}}},1}}$$

10 $$\frac{{\mathrm{d}}{C}_{{{\mathrm{NO}}}}}{{{\mathrm{d}}t}}=\frac{1}{3}\frac{{r}_{{{\mathrm{KS}}}}^{{{\mathrm{NO}}}_3}}{{Y}_{{{\mathrm{KS}}},1}}-\frac{{r}_{{{\mathrm{KS}}}}^{{{\mathrm{NO}}}}}{{Y}_{{{\mathrm{KS}}},2}}-{r}_{{{\mathrm{tr}}}}^{{{\mathrm{NO}}}}$$

11 $$\frac{{\mathrm{d}}{C}_{{{\mathrm{N}}}_{2}{\mathrm{O}}}}{{{\mathrm{d}}t}}=\frac{1}{2}\frac{{r}_{{{\mathrm{KS}}}}^{{{\mathrm{NO}}}}}{{Y}_{{{\mathrm{KS}}},2}}-{r}_{{{\mathrm{tr}}}}^{{{\mathrm{N}}}_{2}{\mathrm{O}}}$$

12 $$\frac{{\mathrm{d}}{X}_{{{\mathrm{KS}}}}}{{{\mathrm{d}}t}}=\mathop{\sum }\limits_{i=1}^{n}{r}_{{{\mathrm{KS}}}}^{\,i}$$

In equations (8)–(12), *Y*_KS,1_ and *Y*_KS,2_ (cells per mmol of Fe(II)) are the growth yields of KS2 for the two successive NDFO reaction steps.

For the case of a mixed *R. ferrooxidans* SW2 and KS incubation (equation (13), in which wKS denotes ‘with KS’), we accounted for toxic inhibition of the growth and activity of photoferrotrophs (*R. ferrooxidans* SW2) by NO. To account for the observed inhibition we incorporated a NO-dependent microbial decay term, which lumps both cell lysis and dormancy in response to NO-related toxic stress. The model considers that the population of active *R. ferrooxidans* SW2 decays rapidly in response to NO exposure:

13 $${\left.\frac{{\mathrm{d}}{X}_{{{\mathrm{SW}}}2}}{{{\mathrm{d}}t}}\right|}_{{{\mathrm{wKS}}}}={r}_{{{\mathrm{SW}}}2}-{k}_{{\mathrm{d}}}\left(1-{f}_{{{\mathrm{tox}}}}^{\;{{\mathrm{NO}}}}\right){X}_{{{\mathrm{SW}}}2}$$

where *k*_d_ (per day) is the first-order decay rate coefficient and ${f}_{{{\mathrm{tox}}}}^{\;{{\mathrm{NO}}}}$ is a concentration-dependent toxicity function (0 < ${f}_{{{\mathrm{tox}}}}^{\;{{\mathrm{NO}}}}$ < 1).

14 $${f}_{{{\mathrm{tox}}}}^{\;{{\mathrm{NO}}}}=\frac{1}{1+{\left(\frac{{C}_{{{\mathrm{NO}}}}}{{K}_{{\mathrm{I}}}^{{{\mathrm{NO}}}}}\right)}^{p}}$$

In equation (14), *C*_NO_ (mM) is the actual aqueous concentration of NO, ${K}_{{\mathrm{I}}}^{{{\mathrm{NO}}}}$ (mM) is the toxic inhibition concentration and *p* is an exponent that characterizes the slope of the curve at the ${K}_{{\mathrm{I}}}^{{{\mathrm{NO}}}}$ inflection point. Toxicity effects were included on the basis of the observation that NO accumulated at detectable levels in the headspace of the reactors and that Fe(II) oxidation stopped after 3.5 days in replicate reactors, despite the lack of a light limitation. Supplementary incubations with different levels of NO confirmed that the activity of *R. ferrooxidans* SW2 is suppressed at the NO concentration of 12 nM (the value used for ${K}_{{\mathrm{I}}}^{{{\mathrm{NO}}}}$ in equation (16)).

Periodic sample collection was simulated as the constant sampling rate, *Q*_s_ (4.62 × 10^−9^ l s^−1^) (based on the total amount of sample volume collected over the duration of the experiment) and was assumed to result in the headspace dilution of NO and N_2_O partial pressures, *p*_NO_ (Pa) and ${p}_{{{\mathrm{N}}}_{2}{\mathrm{O}}}$ (Pa), respectively. Changes in the partial pressures of NO and N_2_O are given by:

15 $$\frac{{\mathrm{d}}{p}_{{{\mathrm{NO}}}}}{{{\mathrm{d}}t}}=-\frac{{p}_{{{\mathrm{NO}}}}{Q}_{{\mathrm{s}}}}{{V}_{{\mathrm{g}}}}+\left(\frac{{V}_{{\mathrm{w}}}}{{V}_{{\mathrm{g}}}}\right){{RT}r}_{{{\mathrm{tr}}}}^{{{\mathrm{NO}}}}$$

16 $$\frac{{\mathrm{d}}{p}_{{{\mathrm{N}}}_{2}{\mathrm{O}}}}{{{\mathrm{d}}t}}=-\frac{{p}_{{{\mathrm{N}}}_{2}{\mathrm{O}}}{Q}_{{\mathrm{s}}}}{{V}_{{\mathrm{g}}}}+\left(\frac{{V}_{{\mathrm{w}}}}{{V}_{{\mathrm{g}}}}\right){{RT}r}_{{{\mathrm{tr}}}}^{{{\mathrm{N}}}_{2}{\mathrm{O}}}$$

In equations (15) and (16), *V*_w_ (l) and *V*_g_ (l) are, respectively, the aqueous and gaseous volumes in the reactors, and $r_{\mathrm{tr}}^{\mathrm{NO}}$ and $r_{\mathrm{tr}}^{\mathrm{N}_{2}{\mathrm{O}}}$ are the mass transfer rates of NO and N_2_O, respectively, as calculated by equation (7).

All model variants were set up as well-mixed batch reactors. Partitioning of NO and N_2_O between the aqueous and gaseous phases was considered in model variants that simulated NDFO. The coupled system of ordinary differential equations was solved in MATLAB using the built-in ordinary differential equation solver ode15s^47^. The *R. ferrooxidans* SW2 and KS growth models were calibrated individually, that is, the parameters for *R. ferrooxidans* SW2 growth were derived by fitting the data from the SW2-only incubations to a model version considering only photoferrotrophy in the absence of KS. Parameters for the growth of KS were derived by fitting the dual-Monod two-step denitrification model to the geochemical data collected from the KS-only incubations. We fitted the logarithms of the parameters rather than the parameters themselves using the lsqnonlin function in MATLAB^48^, thereby alleviating the discrepancy between nominal values that differ by orders of magnitude. Our fitting scheme was based on minimizing the sum of squared differences between the measurements and the simulated output. Calibrated parameter values for photoferrotrophy and NDFO catalysed by the KS culture are presented in Supplementary Table 3 (parameter sensitivity and uncertainty analyses are given in Supplementary Methods 1). The simulation of the combined incubation where KS and *R. ferrooxidans* SW2 were grown together was achieved by combining the parameters fitted from the individual incubations into a lumped model. The only additional parameters in the mixed case were those for the toxicity function. The toxic inhibition concentration for NO, ${K}_{{\mathrm{I}}}^{{{\mathrm{NO}}}}$, was set to the value derived from our toxicity experiments, and the parameters *k*_d_ and *p* were modified to the data in the combined incubation.

### Bioinformatics

Hundreds of thousands of assembly structure report files were downloaded from the RefSeq database at the National Center for Biotechnology Information (NCBI)^49^ to study the distribution of NO reductase genes in bacterial genomes. This number was reduced to ~30,000 assemblies by selecting only the best assembly for each species (as defined in the NCBI taxonomy database information for the genomes^50^). Further analysis was conducted as outlined in Supplementary Method 3.

## Online content

Any methods, additional references, Nature Portfolio reporting summaries, source data, extended data, supplementary information, acknowledgements, peer review information; details of author contributions and competing interests; and statements of data and code availability are available at 10.1038/s41561-024-01560-9.

## Supplementary information

Supplementary Information Supplementary Results 1 and 2, Discussions 1–3, Methods 1–3 and Figs. 1–9.

## Data Availability

Raw data from the incubations, model outputs and further data underpinning the bioinformatics analyses are available via Zenodo at 10.5281/zenodo.13463529 (ref. ^51^).

## Code availability

All model scripts are available via Zenodo at 10.5281/zenodo.13463529 (ref. ^51^).
