# Supplementary material for: Inhibition of phototrophic iron oxidation by nitric oxide in ferruginous environments
Source: Nat Geosci. 2024 Oct 4;17(11):1169–74. doi: 10.1038/s41561-024-01560-9 (PMC11543593; doi:10.1038/s41561-024-01560-9)
Supplement: Supplementary file 1 — Supplementary Results 1 and 2, Discussions 1–3, Methods 1–3 and Figs. 1–9. [file 41561_2024_1560_MOESM1_ESM.pdf]

nature geoscience

Supplementary information https://doi.org/10.1038/s41561-024-01560-9

Inhibition of phototrophic iron oxidation by nitric oxide in ferruginous environments

In the format provided by the authors and unedited

Supplementary Information for:

Inhibition of phototrophic iron oxidation by nitric oxide in ferruginous environments

Verena Nikeleit[1,10], Adrian Mellage[2], Giorgio Bianchini[3], Lea Sauter[1], Steffen Buessecker[4,5], Stefanie Gotterbarm[1], Manuel Schad[1,6], Kurt Konhauser[6], Aubrey L. Zerkle[7], Patricia Sánchez-Baracaldo[3], Andreas Kappler[1,8], Casey Bryce[9*]

Affiliations:

[1]Geomicrobiology, University of Tübingen, Tübingen, Germany

[2] Civil & Environmental Engineering, University of Kassel, Kassel, Germany

[3] School of Geographical Sciences, University of Bristol, Bristol, UK

[4] Department of Earth System Science, Stanford University, Stanford, USA

[5] School of Life Sciences, Arizona State University, Tempe, AZ, USA.

[6] Department of Earth & Atmospheric Sciences, University of Alberta, Edmonton, Canada

[7] Blue Marble Space Institute of Science, Seattle, WA, USA

[8] Cluster of Excellence: EXC 2124: Controlling Microbes to Fight Infections, Tübingen, Germany

[9] School of Earth Sciences, University of Bristol, Bristol, UK

[10] NORCE Norwegian Research Center, Bergen, Norway

*Correspondence:

Dr Casey Bryce, School of Earth Sciences, Wills Memorial Building, Queens Road Bristol, BS8 1RJ

Email: casey.bryce@bristol.ac.uk

Supplementary Results

1. Inhibition of photoferrotrophy by other pathways of nitrate-dependent Fe(II) oxidation

In a further experiment, we observed that inhibition also occurred with the nitrate-reducing Fe(II)-oxidizer *Acidovorax* sp. BoFeN1 (Figure S6). This strain reduces NO_3^- via oxidation of organic carbon. The coupled reaction produces NO_2^-, which abiotically oxidizes Fe(II), with 19% and 29% of the total Fe(II) oxidation likely attributable to abiotic processes (Dopffel et al., 2022). The abiotic reaction between NO_2^- and Fe(II) produces NO and N_2O in a process known as chemodenitrification (Equation 3 - 5; Klueglein et al., 2014). NO_2^- accumulation by *Acidovorax* sp. BoFeN1 is typically in the 1 - 3 mM range under these conditions [1] whereas culture KS typically only sees NO_2^- accumulation up to tens of µM, if at all [2]. When we grew BoFeN1 and SW2 together in a mixed culture, the culture with BoFeN1 alone and the mixed culture showed similar extents of Fe(II) oxidation after 21 days. When SW2 was alone however, the Fe(II) remaining after 21 days was much lower (Figure S6).

$$NO_2 + 2Fe^{2+} + 2H^+ \rightarrow 2Fe^{3+} + NO + H_2O \qquad (1)$$

$$NO + Fe^{2+} + H^+ \rightarrow Fe^{3+} + HNO \qquad (2)$$

$$2HNO \rightarrow N_2O + H_2O \qquad (3)$$

We further evaluated whether NO_2^- itself showed a toxic effect when added to an *R. ferrooxidans* SW2 culture under ferruginous conditions, and observed no Fe(II) oxidation attributable to *R. ferrooxidans* SW2 above 10 µM NO_2^- (Figure S8). However, in the absence of Fe(II), and with acetate as the electron donor, *R. ferrooxidans* SW2 tolerated NO_2^- concentrations above 500 µM (Figure S6), and did not exhibit growth inhibition until 2mM NO_2^-. This strongly suggests that the abiotic reaction of NO_2^- and Fe(II), which yields NO (and HNO which is also potentially toxic; Fang. 2004) as an intermediate [3], drives the increased

sensitivity to NO_2^- under ferruginous conditions observed in these experiments, not NO_2^- itself which can be tolerated at much higher concentrations in the absence of Fe(II).

2. Results of comparative genomics

In order to visualize how genetic capabilities for NO detoxification in our cultured phototrophs compared to other phototrophic bacteria, we implemented comparative genomic analysis to map the presence of *norV*, *norB* or *hmpA* across all phototroph genomes available in the NCBI RefSeq database at the time of our analysis (June 2020). Phototrophs were identified based on photosynthetic marker genes encoding for cyanobacterial photosystem I (*psaB*) and II (*psbA*) and non-cyanobacterial type I and type II reaction centres (*pshA/pscA* and *pufL* respectively). Marker genes for NO reduction are widespread within the phototroph genomes: 53 (7%) have *hmpA*, 314 (41%) have *norV* and 129 (17%) have *norB* (Extended Data Figure 7). Anoxygenic phototrophs with type I reaction centres (i.e., the *Chlorobi*) and *Cyanobacteria* almost all contain *norV* (98% of *Cyanobacteria*, n = 259; 92% of *Chlorobi*, n = 22) and none contain *hmpA*. None of the *Chlorobi* have *norB* genes whilst a small number of *Cyanobacteria* (28, 11%) do. *norV* is much less common in anoxygenic phototrophs with type II reaction centres (i.e., purple bacteria), as it is only present in 36 genomes (8%), however *norB* (102, 21%) and *hmpA* (53, 11%) genes are more common.

Supplementary Discussion

1. Would these interactions be expected in modern environments?

The Fe(II) concentrations used in this study are high compared to modern environments and are at the very upper end of estimates for Archean [4] and Proterozoic ferruginous oceans [5,6], although in reality seawater Fe(II) concentrations at the time of BIF deposition were likely lower (up to 0.5 mM; Morris, 1993). The NO_3^- concentrations are also higher than would be

expected in both modern and ancient settings. The Archean ocean is thought to have contained < 1μM NO_3^- [8] whereas modern surface NO_3^- concentrations range from e.g. 25 µM to < 0.1 µM in the Indian Ocean or 8 µM to < 0.1 µM in the equatorial Pacific [9]. However, it must be considered that measured natural NO_3^- concentrations are highly variable and often low because they reflect high N turnover and not low NO_3^- availability. Indeed, Archean NO_3^- and NO_2^- concentrations are estimated to be low precisely because of their reaction with ferrous iron (as well as reaction with HS^- and other reduced sulfur species), which produces short-lived reactive N species such as NO (Stüeken *et al.*, 2016).

It is important to stress that the high Fe(II) (10 mM) and NO_3^- (0.4 and 1 mM) concentrations used here were a practical necessity to quantify the co-culture dynamics experimentally as the Fe(II):NO_3^- ratio has to be set such that there is enough NO_3^- to observe Fe(II) oxidation by culture KS, but not so much as to leave no remaining Fe(II) for the photoferrotroph. 0.4 mM NO_3^- is the lowest concentration we could use whilst still being able to clearly measure the Fe(II) oxidation. However, our concentrations are in line with similar experimental studies [6,10]. Because NO is the driving force of inhibition it is unlikely that the excess of Fe(II) has a significant effect on whether or not inhibition occurs. Moreover, we observe that concentrations as low as 12 nM NO have the ability to hinder Fe(II) oxidation (Figure S13 and Figure S14). Therefore, we expect that environmentally realistic NO concentrations can exert a strong selective pressure on the microbial community in modern ferruginous environments such as sediments, oxygen minimum zones and ferruginous, stratified lakes. Previous DNA and cultivation-based studies had shown the potential for nitrate-reducing Fe(II)-oxidizers and photoferrotrophs to co-exist in the upper layers of both freshwater and marine sediments, a zone which is only a few millimetres thick (Laufer et al., 2016), as well as on larger meter scales in stratified lakes (Michiels et al., 2017). Our results suggest that true co-existence is likely challenging for the photoferrotrophs. However, they

may have to live below the zone of active nitrate-reduction in regions where nitrate-reduction and production of toxic reactive nitrogen species is relatively high. Where this zone extends below the photic zone they would be excluded, however a niche may exist for them in regions where light penetration is deeper.

2. ***Could nitrate-dependent Fe(II) oxidation compensate for BIF deposition by photoferrotrophs?***

If photoferrotrophs were inhibited, how much BIF deposition could be driven by nitrate-reducing Fe(II) oxidation alone? It has been previously hypothesized that the inverse correlation between $\delta^{15}N$ and $\delta^{13}C_{carb}$ values in the Palaeoproteozoic Brockman IF could be explained by partial denitrification by nitrate-reducing Fe(II)-oxidizing bacteria, with the NO_3^- sourced from nitrification (oxidation of NH_4^+) in a stratified water column [11]. It is estimated that deposition of the 2.48 Ga Dales Gorge Member of the Brockman Iron Formation required peak Fe(II) oxidation rates of 7.85×10^{11} mol of Fe per year [12]. Thus, given the stoichiometry of Fe(II):NO_3^- in the nitrate-reducing Fe(II)-oxidizing reaction (5:1; Equation 2), reduction of 1.57×10^{11} mol of NO_3^- per year would be required to oxidize all of this iron. Primary productivity in the Archean and Paleoproterozoic could have been as low as 10% of modern levels [13], with a further 50% decrease in N_2 fixation rates due to Mo limitation of nitrogenase enzymes [14], yielding an estimated nitrogen fixation rate of 5×10^{11} mol/year. 31% of this fixed nitrogen would need to be oxidized to provide enough NO_3^- to form adequate amounts of BIF from nitrate-reducing Fe(II) oxidation, assuming no NO_3^- loss via heterotrophic denitrification or anaerobic ammonium oxidation. Given that there is no persistent signal for heterotrophic denitrification or anammox in the $\delta^{15}N$ record until ~2.3 Ga [15], and that Fe^{2+} could have been a much more widely available electron donor in deep ferruginous oceans than organic matter, we find this to be a plausible assumption. These estimates of N availability do not account for abiotic sources of fixed N such as lightning, volcanism, and bolide impacts which could

collectively account for N fluxes of the same magnitude as biological N fixation (~ 2.5 x 10^11 mol N yr^{-1}) [16–18] and would make the potential for nitrate-reducing Fe(II) oxidation even more widespread. Therefore, we hypothesize that it was possible that nitrate-reducing Fe(II) oxidation could have compensated to a significant extent for BIF deposition after photoferrotrophs became inhibited.

3. *Could heightened NO influence other microbial lineages?*

Whilst our study has focused on the effect of nitrate-reducing Fe(II) oxidation on anoxygenic phototrophs, other microbial lineages could also be affected by NO accumulation under ferruginous conditions. Indeed previous authors have noted that early accumulation of NO via photochemical reactions in the atmosphere would have provided one of the most promising electron acceptors for early life [19–21]. It is even considered likely that NO and O_2 reductases share an evolutionary history [22,23]. Local accumulation of NO in the early oceans has also been proposed to have led to antagonistic interactions between denitrifying (e.g., NO-producing) bacteria and cyanobacteria [24]. Those authors even go so far as to suggest that NO toxicity would represent the first cyanobacterial "disease" which gave rise to NO defense mechanisms in cyanobacteria and laid the groundwork for the evolution of phytopathogenesis. The ubiquity of NO detoxification genes we observe in the *Chlorobi* and Cyanobacteria may hint towards this shared stress early in each group's evolutionary history.

Supplementary Figures:

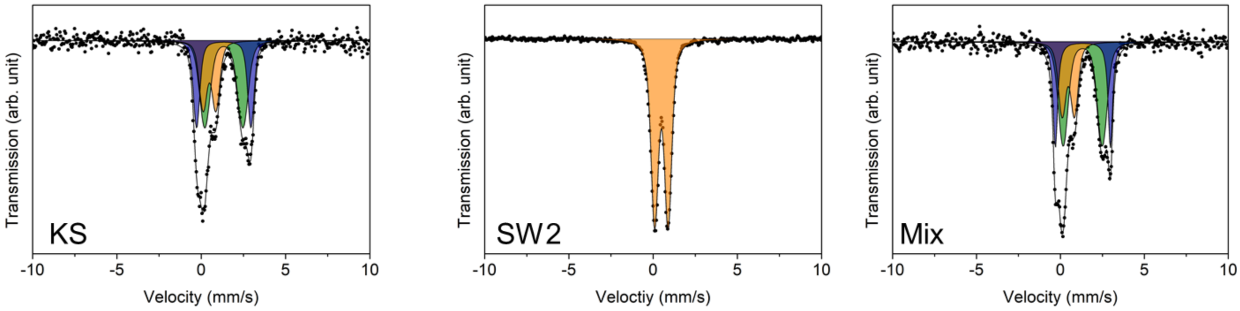

Supplementary Figure S1: Moessbauer spectra of minerals formed by KS (left), *R. ferrooxidans* **SW2 (middle) and the mixed incubation of both strains (right).** Closed circles represent collected data, while the solid black line represents the data fit. Orange shaded areas represent a short-range ordered (SRO) Fe(III) mineral; the green shaded area siderite; and the blue shaded area a second Fe(II) mineral phase, possibly vivianite. *R. ferrooxidans* SW2 exclusively formed a SRO Fe(III) mineral, possibly ferrihydrite. Both, the KS and mixed incubations contain Fe(II) as well as Fe(III) mineral phases.

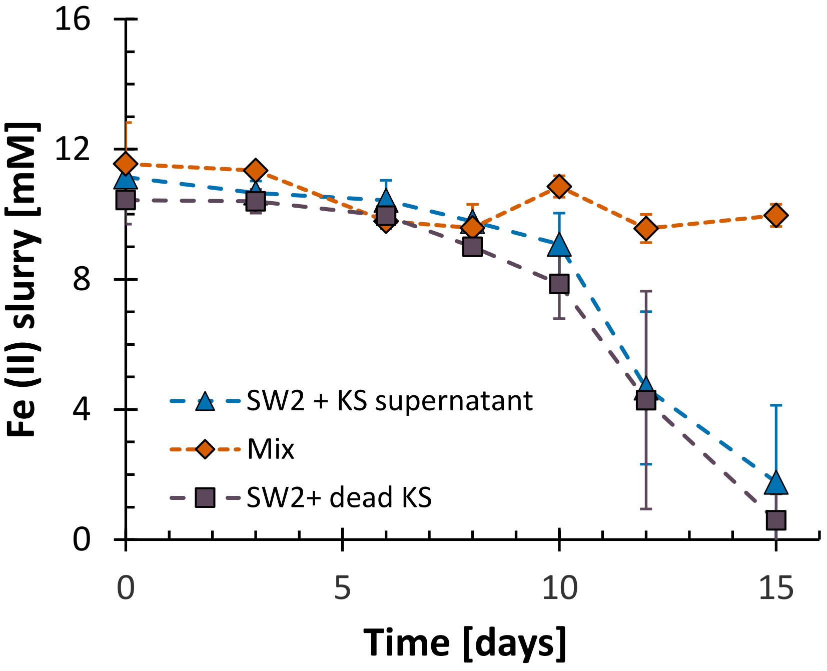

Supplementary Figure S2. Fe(II) oxidation is complete when *R. ferrooxidans* SW2 is combined with dead KS cells (autoclaved prior to inoculation) or with the supernatant from the KS culture (filtered prior to inoculation). Inhibition still occurs when live KS cells are added. Errors are the standard deviation of biological triplicates.

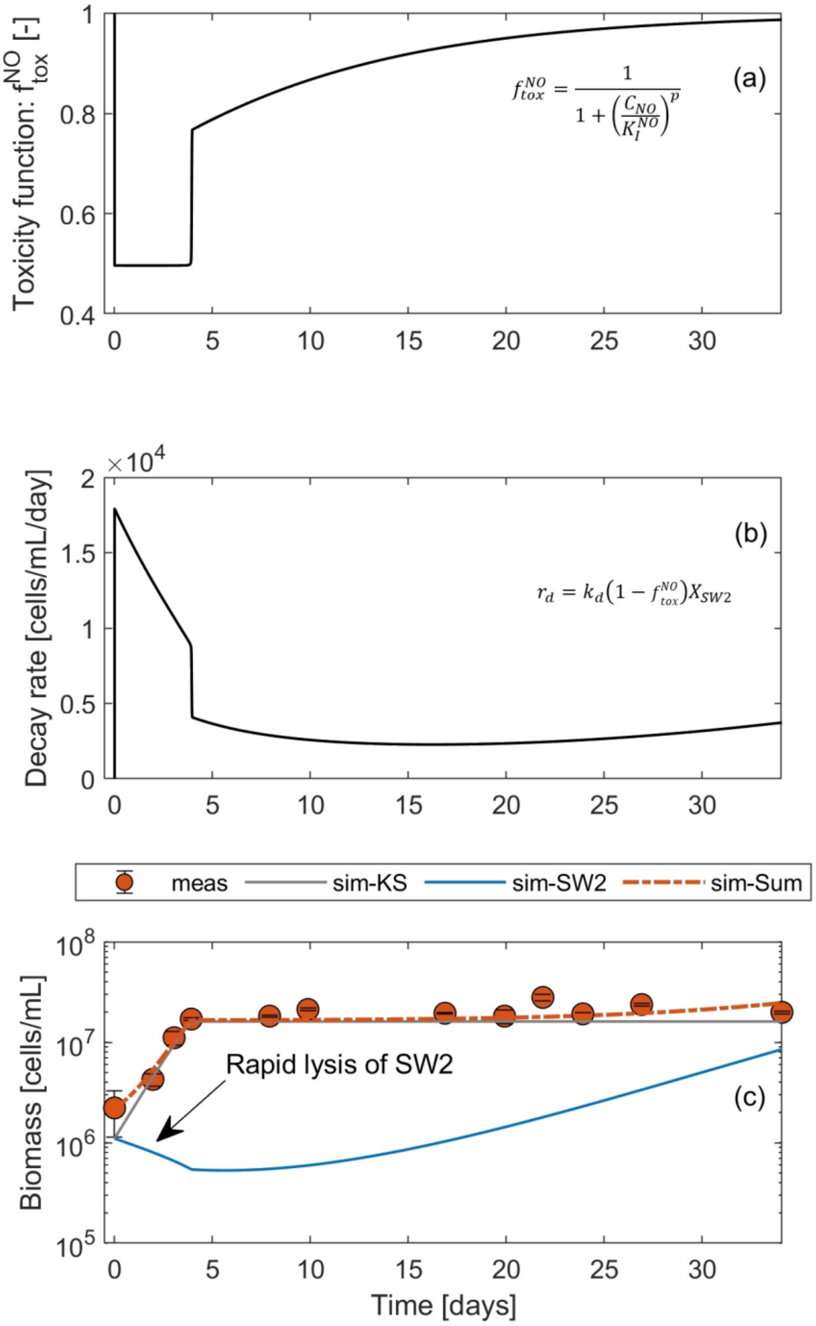

Supplementary Figure S3. Simulated toxicity-induced cells lysis (decay) during the combined SW2+KS incubation. The toxicity function (a) drops to values below 0.5 during the period of NO production fueling the decay of SW2 cells (c). The decay rate is plotted in panel (b). Once the concentration of NO drops, the toxicity function approaches 1 and the SW2 population begins to recover.

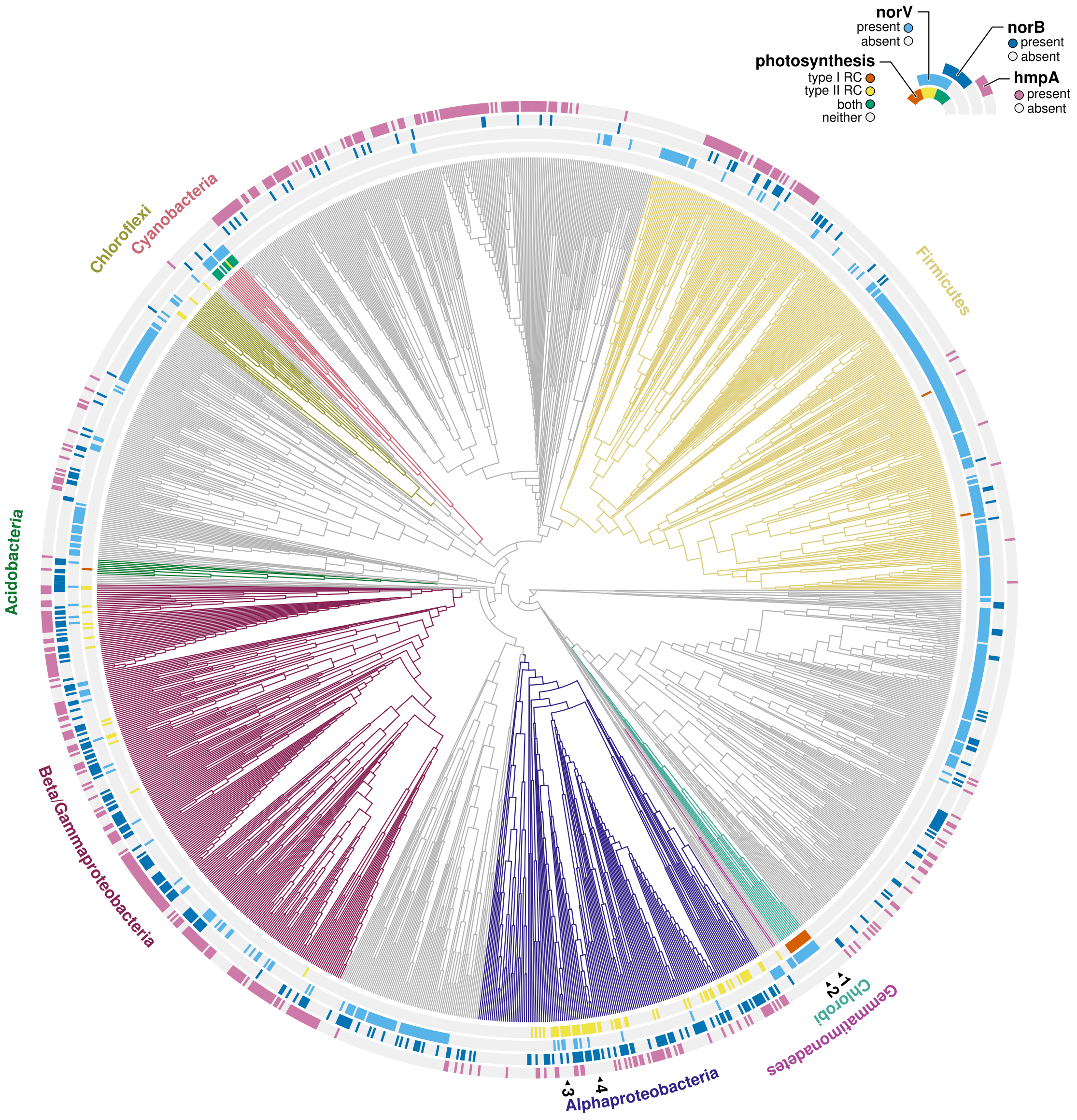

Supplementary figure S4: Distribution of phototrophy and nitric oxide detoxification genes across 28,413 bacterial genomes. Numbers denote positions of the 4 photoferrotrophs cultured in this study: (1) *Chlorobium ferrooxidans* KoFox, (2) *Chlorobium* sp. N1, (3) *Rhodovulum iodosum*, and (4) *Rhodobacter ferrooxidans* SW2.

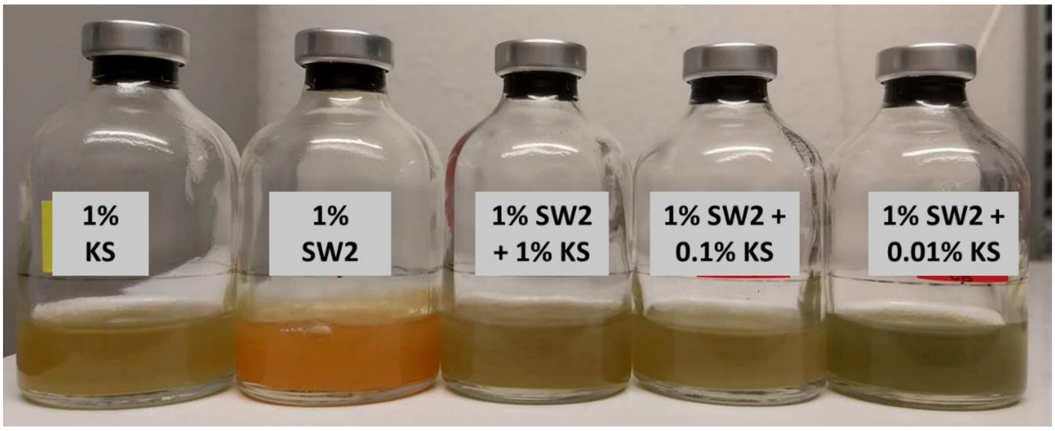

Supplementary Figure S5: Fe(II) oxidation in mixed cultures of SW2 and KS with varying inoculum ratios. This demonstrates that the ability of SW2 to complete Fe(II) oxidation was independent of the initial amount of KS cells added.

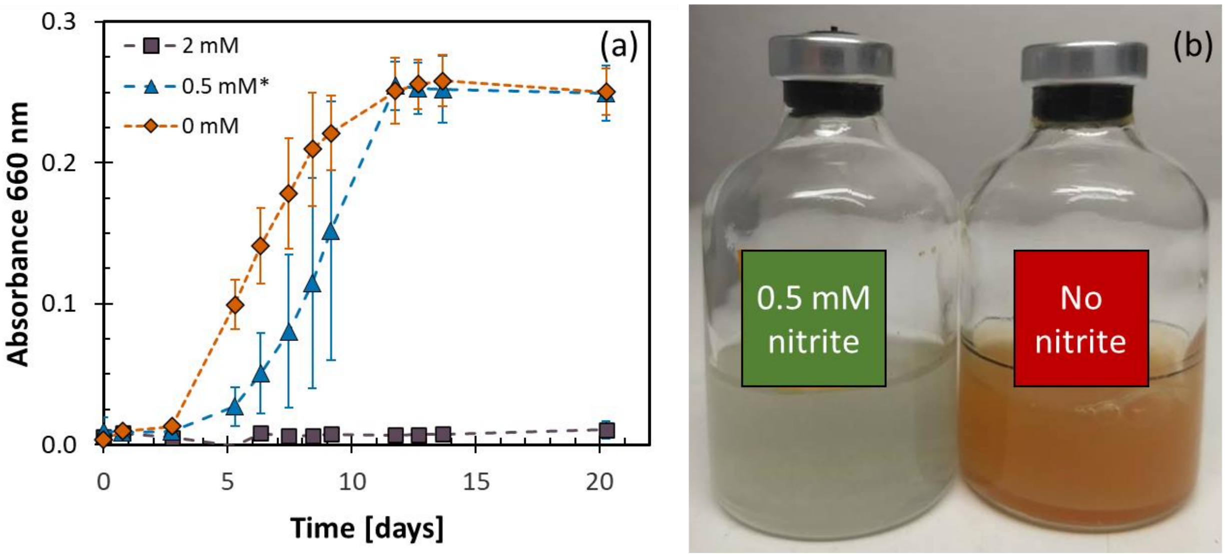

Supplementary Figure S6. A: Growth of *R. ferrooxidans* SW2 cells (measured by absorbance at 660 nm) in the presence of different concentrations of nitrite and 5mM acetate in the absence of Fe(II). * The 0.5 mM nitrite set up represents measurements from biological duplicates where the error shows the range of the two bottles. All others are from biological triplicates with error derived from the standard deviation of biological triplicates. B: Cultures of *R. ferrooxidans* SW2 in the presence and absence of nitrite with 10 mM Fe(II).

Supplementary Tables

Table S1: Hyperfine parameters of the mineral products of setups KS, *R. ferrooxidans* **SW2 and Mix incubation. Results of the fitting spectra.** δ – center shift, ΔEQ – quadrupole splitting, R.A. – relative abundance of the mineral phase at the given temperature, ± - error in the relative abundance, χ2 indicates the goodness of fit. [1] [25], [2] [26], [3] [27].

Sample	Temp [K]	Phase	δ [mm s^{-1}]	ΔE_Q [mm s^{-1}]	R. A. [%]	±	χ^2
KS	77	Siderite[1]	1.34	2.26	43.9	8.2	0.62
		Ferrihydrite[2]	0.49	0.76	26.7	4.5	
		Vivianite[3]	1.33	3.22	29.3	8.6	
SW2	77	Ferrihydrite[2]	0.49	0.80	100	-	1.58
Mix	77	Siderite[1]	1.33	2.33	47.1	3.7	0.53
		Ferrihydrite[2]	0.47	0.73	26.1	2.3	
		Vivianite[3]	1.34	3.28	26.8	3.8	

Table S2. Medium composition used for experiments with strains other than *R. ferrooxidans* SW2

Strain	Reference	Growth media
Rhdovulum iodosum	Straub et al. (1999)	22 mM bicarbonate-buffered media containing following salts:<br>26.4 g L^{-1} NaCl, 6.8 g L^{-1} $MgSO_4.7H_20$, 5.7 g L^{-1} $MgCl_2.6H_2O$, 1.5 g L^{-1} $CaCl_2.2H_2O$, 0.66 g L^{-1} KCl, 0.09 g L^{-1} KBr 0.4 g L^{-1} KH_2PO_4, 0.25 g L^{-1} NH_4Cl<br><br>Additives: 1 mL L^{-1} sterile filtered 7-vitamin solution (Widdel and Pfennig, 1981), trace element solution (Widdel et al., 1983) and selenite-tungstate solution (Widdel and Bak, 1992)<br><br>pH: 7.0<br><br>Added substrates: 10 mM $FeCl_2$
Chlorobium sp. N1	Laufer et al. (2016)	22 mM bicarbonate-buffered media containing following salts:<br>17.3 g L^{-1} NaCl, 0.025 g L^{-1} $MgSO_4.7H_20$, 8.6 g L^{-1} $MgCl_2.6H_2O$, 0.99 g L^{-1} $CaCl_2.2H_2O$, 0.39 g L^{-1} KCl, 0.059 g L^{-1} KBr, 0.05 g L^{-1} KH_2PO_4, 0.25 g L^{-1} NH_4Cl<br><br>Additives: 1 mL L^{-1} sterile filtered 7-vitamin solution (Widdel and Pfennig, 1981), trace element solution (Widdel et al., 1983) and selenite-tungstate solution (Widdel and Bak, 1992)<br><br>pH: 7.0<br><br>Added substrates: 10 mM $FeCl_2$
Acidovorax sp. BoFeN1		22 mM bicarbonate-buffered media containing following salts:<br><br>0.6 g/L KH_2PO_4, 0.3 g/L NH_4Cl, 0.025 g/L $MgSO_4{\cdot}7\ H_2O$, 0.4 g/L $MgCl_2{\cdot}6\ H_2O$, 0.1 g/L $CaCl_2{\cdot}2\ H_2O$<br>Additives: 1 mL L^{-1} sterile filtered 7-vitamin solution (Widdel and Pfennig, 1981), trace element solution (Widdel et al., 1983) and selenite-tungstate solution (Widdel and Bak, 1992)<br><br>pH: 7.0<br><br>Added substrates: 10 mM $FeCl_2$, 2 mM NO_3^-, 0.5 mM sodium acetate

Supplementary Method 1: Parameter uncertainty and sensitivity analysis

Relative parameter uncertainties were estimated via a linearized uncertainty analysis on the log-transformed parameters and are reported on the matrix-diagonal in Figure S15, along with the correlation coefficients of log-parameter uncertainties. In addition, results from a linearized sensitivity analysis obtained via the automated model calibration (of the log-parameter values) procedure are presented in Figures S12 and S13 for the SW2 and KS-only incubations.

The model formulation presented herein differs from previous models that simulated NDFO, namely, Jamieson et al. (2018) and Huang et al. (2023), in particular, with regards to the number of denitrification steps considered. Nonetheless, both sets (for NO_3^- and NO) of calibrated electron acceptor and donor half saturation coefficients fall within the range of coefficients calibrated in by previous models (e.g. Jamieson et al 2018 and Huang et al 2023). In both of those models, the half-saturation constant for iron was orders of magnitude higher than those of nitrogen species, analogous to the fitter parameter set herein. Moreover, our specific growth rate constants ($\boldsymbol{\mu_{max}}$) fall within the range of previously reported denitrification parameters (e.g., Almeida et al., 1995; Schreiber et al., 2009; Ni et al., 2011), albeit coupled to organic carbon as the electron donor.

The yields for NDFO, $\boldsymbol{Y_{KS,1}}$ and $\boldsymbol{Y_{KS,2}}$, exhibit the lowest relative uncertainty and their uncertainties are not correlated with that of other parameters. The yields, together with $\boldsymbol{\mu_{max}^{NO3}}$, $\boldsymbol{K_{NO}}$ and $\boldsymbol{K_{Fe(II)}^{NDFO}}$ are the parameters that the model for KS is most sensitive to. These are also the parameters with the lowest relative uncertainties, albeit only those of $\boldsymbol{\mu_{max}^{NO3}}$ and the yields are low enough to provide confidence in the absolute parameter value. The high correlation between the uncertainties of parameters such as $\boldsymbol{\mu_{max}^{NO}}$ and $\boldsymbol{K_{NO}}$suggests that, for several parameters, the model and current dataset can only be used to reliably estimate their ratio and not their absolute value. A similar strong correlation can be observed between $\boldsymbol{\mu_{max}^{ph}}$ and $\boldsymbol{K_{Fe}^{ph}}$ in the SW2 incubation suggesting that, for this particular experiment, the model can also only reliably determine the ratio of both parameters and not their absolute value. Thereby, implying that the Monod-expression is effectively in the first-order range. Nonetheless, the current parameter set accurately predicts the behavior of the system. The overall root mean squared error (RMSE) for each model was computed by considering the difference between

measured and simulated values for all data-types, normalized by each measurement's standard deviation. In addition, we computed RMSE values for each data type to highlight the model accuracy for each measurement. All data-specific RMSE values fall within measurement standard deviation bounds.

Table S3. Calibrated parameter values and goodness-of-fit, reported as root mean squared error (RMSE) between simulated and measured values, for both pure-culture incubations of either phototrophs or KS.

Phototrophs (SW2)					
Parameter	Value	Units	RMSE[1]		
μ_{max}^{ph}	1	[day^{-1}]	*Overall*	0.014	[-]
$K_{Fe(II)}^{ph}$	32.7	[mM]	*Fe(II)*	0.92	[mM]
Y_{SW2}	1.28×10^{10}	[cells mmol$_{Fe(II)}^{-1}$]	*Biomass*	2.6×10^{6}	[cells mL^{-1}]

KS-culture					
Parameter	Value	Units	RMSE[1]		
μ_{max}^{NO3}	1.18	[day^{-1}]	*Overall*	8.09	[-]
μ_{max}^{NO}	126	[day^{-1}]	*Fe(II)*	1.07	[mM]
K_{NO3}	2.47×10^{-3}	[mM]	*NO_3^-*	0.15	[mM]
K_{NO}	1.61×10^{-2}	[mM]	*pNO*	0.06	[Pa]
$K_{Fe(II)}^{NDFO}$	7.50	[mM]	*pN_2O*	18.8	[Pa]
$Y_{KS,1}$	4.6×10^{9}	[cells mmol$_{Fe(II)}^{-1}$]	*Biomass*	7.9×10^{5}	[cells mL^{-1}]
$Y_{KS,2}$	1.2×10^{9}	[cells mmol$_{Fe(II)}^{-1}$]			
k_{tr}	16.9	[day^{-1}]			
Toxicity parameters for combined (SW2+KS) case:					
k_d	9.0×10^{-6}	[day^{-1}]			
K_I^{NO}	1.2×10^{-5}	[mM]			
p	0.15	[-]			

[1]RMSE values are reported as overall values, normalized by the measurement standard deviations, or as absolute values per data-type (denoted by italicized text).

Supplementary Method 2: Parameter uncertainty and sensitivity analysis

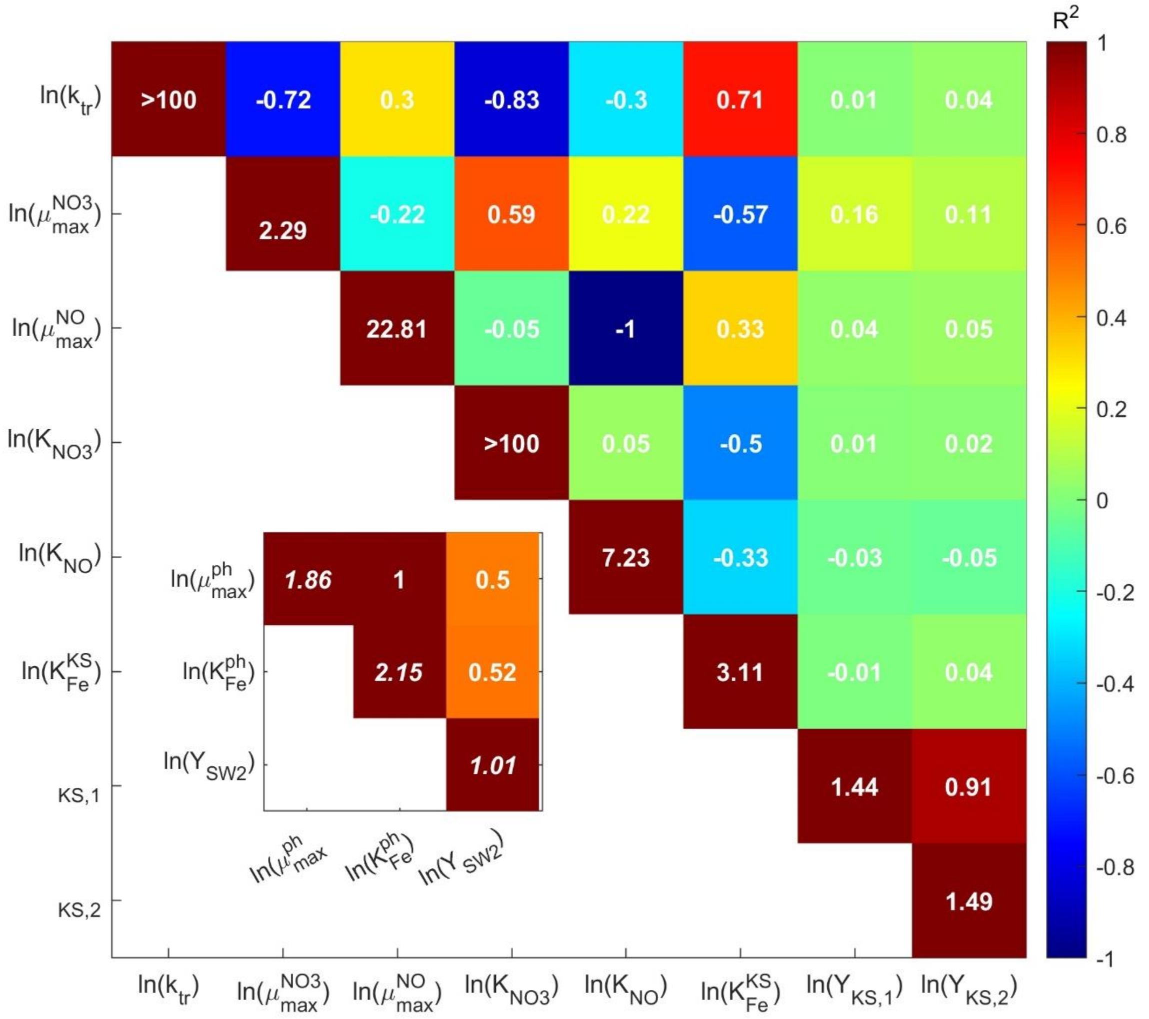

Supplementary Figure S7. Parameter uncertainty correlation matrix for KS- and SW2-only incubations, large and small panels, respectively. The upper triangle (of each panel) shows correlation coefficients of log-parameter uncertainties. The relative errors (×/÷) for each parameter are shown on the diagonal. Parameters with a relative error close to 1 have a low uncertainty.

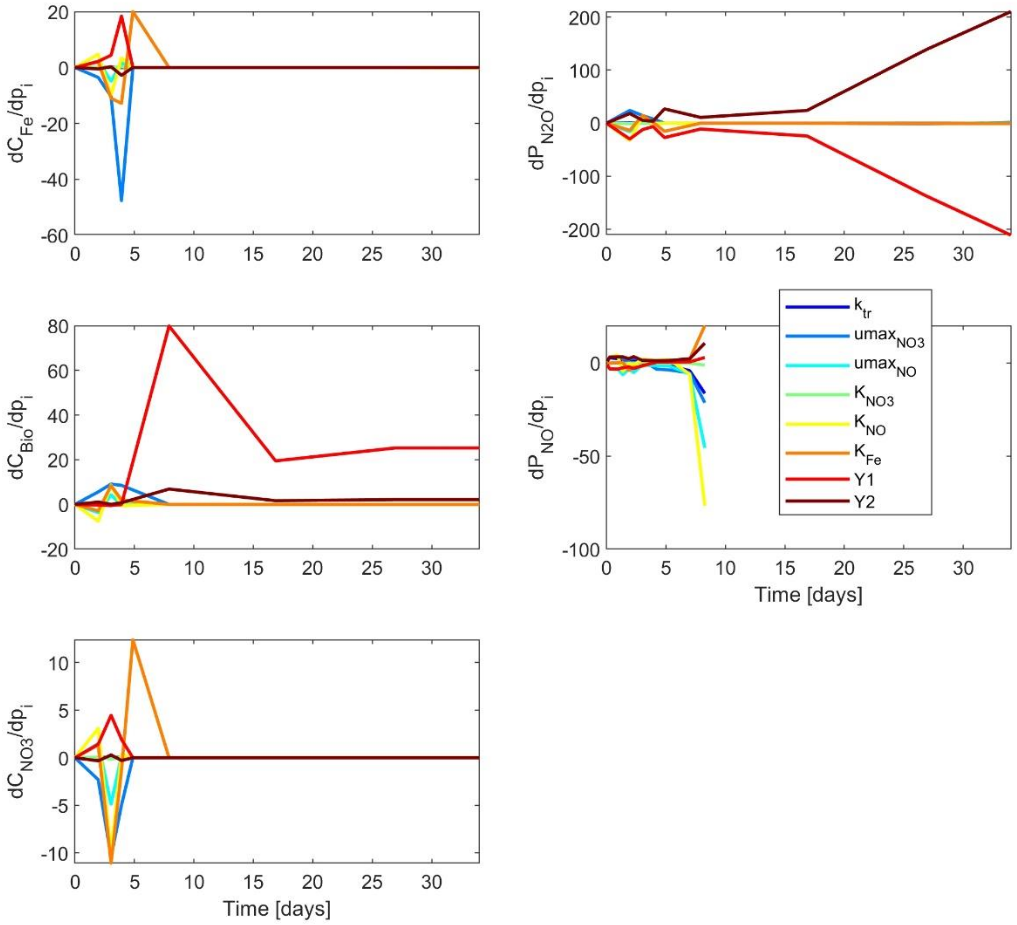

Supplementary Figure S8. Parameter sensitivities plotted at each measurement time point during the KS-only incubation, where i denotes the i-th parameter, listed in order in the figure caption.

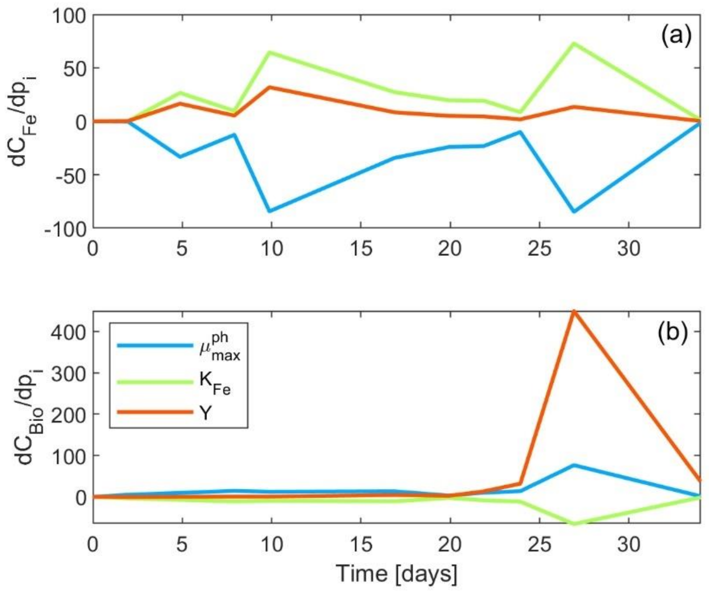

Supplementary Figure S9. Parameter sensitivities plotted at each measurement time point during the SW2-only incubation, where i denotes the i-th parameter, listed in order in the figure caption.

Supplementary Method 3: Comparative genomics analysis

RNAmmer v1.2 was used to obtain SSU rRNA sequences from each genome [31]. Genomes without any 16S sequence were excluded from the analysis, with a final dataset size of 28,413 genomes.

An SSU rRNA phylogenetic tree was used to illustrate and map the presence of nitric oxide reductase genes in bacterial genomes. To build this, 16S sequences were aligned with MAFFT v7.471 [32] using as a guide tree the topology of the NCBI taxonomy in which nodes with fewer than or equal to 2000 terminal descendants were transformed in polytomies. Sequences descending from each node were aligned separately and the alignments were then merged. Using the complete 16S alignment, the % identity was estimated amongst the 403,635,078 possible pairs of genomes.

A maximum-likelihood tree was built using IQTREE v2.1.2 [33], with the guide tree as a constraint. The topology obtained contains all major relevant bacterial groups, however deep-

branching relationships do not reflect evolutionary histories; the tree was rooted arbitrarily using the *Deferribacteres* as an outgroup.

Genomes were screened using BLAST searches (version 2.11.0; Camacho et al., 2009) to assess whether each contained nitric oxide reductase and photosynthesis genes. Phylogenetic gene trees for each gene were built to assess orthology.

For photosynthesis [35] these genes were used as markers: *psaB* (photosystem I), *pshA* (type I reaction centres), *psbA* (photosystem II), *pufL* (type II reaction centres). For NO reductase, these genes were screened: *norV* (Shimizu et al., 2015), *norB* (i.e. the *cnorB* gene, which encodes for a protein that reduces NO using cytochromes as electron donors, and the *qnorB* gene, which encodes for a protein that uses electrons from quinol (Braker and Tiedje, 2003)), and *hmpA* (encoding a flavohemoglobin implicated in NO detoxification; [38,39]). Query sequences for all genes above and their accession number are found in Supplementary File S1.

To obtain a better readability of the tree, the branches in Extended Data Figure 7 and Figure S4 were collapsed based on a 16S % identity threshold. In Extended Data Figure 7 we used a threshold of 97%, while in Figure S4 we used a threshold of 90%. Additionally, in Extended Data Figure 7 all non-photosynthetic strains were pruned from the tree. In all figures, the tips representing the four strains that were cultured in this study were always kept as individual tips, regardless of their identity score. Supplementary information for mapping of nitric oxide detoxification abilities in bacteria, particularly in phototrophs (included as attached excel files).

Tables showing the presence and absence of genes across the genomes queried are available at https://zenodo.org/records/13463529.

Supplementary References

1. Klueglein, N. *et al.* Potential role of nitrite for abiotic Fe(II) oxidation and cell encrustation during nitrate reduction by denitrifying bacteria. *Applied and Environmental Microbiology* **80**, 1051–1061 (2014).
2. Tominski, C., Heyer, H., Lösekann-Behrens, T., Behrens, S. & Kappler, A. Growth and population dynamics of the anaerobic Fe(II)-oxidizing and nitrate-reducing enrichment culture KS. *Appl Environ Microbiol* **84**, 1–15 (2018).
3. Kampschreur, M. J., Kleerebezem, R., de Vet, W. W. J. M. & Van Loosdrecht, M. C. M. Reduced iron induced nitric oxide and nitrous oxide emission. *Water Research* **45**, 5945–5952 (2011).
4. Thompson, K. J. *et al.* Photoferrotrophy, deposition of banded iron formations, and methane production in Archean oceans. *Sci Adv* **5**, 1–9 (2019).
5. Derry, L. Causes and consequences of mid-Proterozoic anoxia. *Geophysical Research Letters* **42**, 8538–8546 (2015).
6. Stanton, C. L. *et al.* Nitrous oxide from chemodenitrification: A possible missing link in the Proterozoic greenhouse and the evolution of aerobic respiration. *Geobiology* **16**, 597–609 (2018).
7. Morris, R. C. Genetic modelling for banded iron-formation of the Hamersley Group, Pilbara Craton, Western Australia. *Precambrian Research* **60**, 243–286 (1993).
8. Ranjan, S., Todd, Z. R., Rimmer, P. B., Sasselov, D. D. & Babbin, A. R. Nitrogen Oxide Concentrations in Natural Waters on Early Earth. *Geochemistry, Geophysics, Geosystems* **20**, 2021–2039 (2019).
9. Altabet, M. A. & Francois, R. Sedimentary nitrogen isotopic ratio as a recorder for surface ocean nitrate utilization. *Global Biogeochemical Cycles* **8**, 103–116 (1994).
10. Thompson, K. J. *et al.* Photoferrotrophy, deposition of banded iron formations, and methane production in Archean oceans. *Science Advances* **5**, 1–9 (2019).
11. Busigny, V., Lebeau, O., Ader, M., Krape, B. & Bekker, A. Nitrogen cycle in the Late Archean ferruginous ocean. *Chem Geol* **362**, 115–130 (2013).
12. Konhauser, K. O. *et al.* Phytoplankton contributions to the trace-element composition of Precambrian banded iron formations. *Bulletin of the Geological Society of America* **130**, 941–951 (2018).
13. Canfield, D. E., Glazer, A. N. & Falkowski, P. G. The evolution and future of earth's nitrogen cycle. *Science* **330**, 192–196 (2010).
14. Zerkle, A. L., House, C. H., Cox, R. P. & Canfield, D. E. Metal limitation of cyanobacterial N2 fixation and implications for the Precambrian nitrogen cycle. *Geobiology* **4**, 285–297 (2006).
15. Zerkle, A. L. *et al.* Onset of the aerobic nitrogen cycle during the Great Oxidation Event. *Nature* 1–10 (2017) doi:10.1038/nature20826.
16. Harman, C. E. *et al.* Abiotic O 2 Levels on Planets around F, G, K, and M Stars: Effects of Lightning-produced Catalysts in Eliminating Oxygen False Positives . *The Astrophysical Journal* **866**, 56 (2018).
17. Kasting, J. F. Bolide impacts and the oxidation state of carbon in the Earth's early atmosphere. *Origins of Life and Evolution of the Biosphere* **20**, 199–231 (1990).
18. Mather, T. A., Pyle, D. M. & Allen, A. G. Volcanic source for fixed nitrogen in the early Earth's atmosphere. *Geology* **32**, 905–908 (2004).
19. Wong, M. L., Charnay, B. D., Gao, P., Yung, Y. L. & Russell, M. J. Nitrogen Oxides in Early Earth's Atmosphere as Electron Acceptors for Life's Emergence. *Astrobiology* **17**, 975–983 (2017).
20. Hu, Z., Wessels, H. J. C. T., van Alen, T., Jetten, M. S. M. & Kartal, B. Nitric oxide-dependent anaerobic ammonium oxidation. *Nature Communications* **10**, (2019).
21. Ducluzeau, A. L. *et al.* Was nitric oxide the first deep electron sink? *Trends in Biochemical Sciences* **34**, 9–15 (2009).
22. Chen, J. & Strous, M. Denitrification and aerobic respiration, hybrid electron transport chains and co-evolution. *Biochimica et Biophysica Acta* **1827**, 136–144 (2013).
23. Saraste, M. & Castresana, J. Cytochrome oxidase evolved by tinkering with denitrification enzymes. *FEBS Letters* **341**, 1–4 (1994).
24. Santana, M. M., Gonzalez, J. M. & Cruz, C. Nitric Oxide Accumulation : The Evolutionary Trigger for Phytopathogenesis. *Frontiers in microbiology* **8**, 1–13 (2017).
25. Forester, D. & Koon, N. Mössbauer investigation of metamagnetic FeCO3. *Journal of Applied Physics* **40**, 1316–1317 (1969).
26. Eickhoff, M. *et al.* Nickel partitioning in biogenic and abiogenic ferrihydrite: The influence of silica and implications for ancient environments. *Geochimica et Cosmochimica Acta* **140**, 65–79 (2014).
27. Gonser, U. & Grant, P. Determination of Spin Directions and Electric Field Gradient Axes in Vivianite by Polarized Recoil-Free γ-Rays. *physica status solidi (b)* **21**, 331–342 (1967).
28. Almeida, J. S., Julio, S. M., Reis, M. A. M. & Carrondo, M. J. T. Nitrite inhibition of denitrification by Pseudomonas fluorescens. *Biotechnology and bioengineering* **46**, 194–201 (1995).

29. Schreiber, F., Loeffler, B., Polerecky, L., Kuypers, M. M. & De Beer, D. Mechanisms of transient nitric oxide and nitrous oxide production in a complex biofilm. *The ISME journal* **3**, 1301–1313 (2009).
30. Ni, B., Ruscalleda, M., Pellicer-Nacher, C. & Smets, B. F. Modeling nitrous oxide production during biological nitrogen removal via nitrification and denitrification: extensions to the general ASM models. *Environmental Science & Technology* **45**, 7768 (2011).
31. Lagesen, K. *et al.* RNAmmer: Consistent and rapid annotation of ribosomal RNA genes. *Nucleic Acids Research* **35**, 3100–3108 (2007).
32. Katoh, K. & Standley, D. M. MAFFT multiple sequence alignment software version 7: Improvements in performance and usability. *Molecular Biology and Evolution* **30**, 772–780 (2013).
33. Nguyen, L. T., Schmidt, H. A., Von Haeseler, A. & Minh, B. Q. IQ-TREE: A fast and effective stochastic algorithm for estimating maximum-likelihood phylogenies. *Molecular Biology and Evolution* **32**, 268–274 (2015).
34. Camacho, C. *et al.* BLAST+: Architecture and applications. *BMC Bioinformatics* **10**, 1–9 (2009).
35. Cardona, T. A fresh look at the evolution and diversification of photochemical reaction centers. *Photosynthesis Research* **126**, 111–134 (2015).
36. Shimizu, T., Hirai, S., Yokoyama, E., Ichimura, K. & Noda, M. An evolutionary analysis of nitric oxide reductase gene norV in enterohemorrhagic Escherichia coli O157. *Infection, Genetics and Evolution* **33**, 176–181 (2015).
37. Braker, G. & Tiedje, J. M. Nitric oxide reductase (norB) genes from pure cultures and environmental samples. *Applied and Environmental Microbiology* **69**, 3476–3483 (2003).
38. Forrester, M. T. & Foster, M. W. Protection from nitrosative stress: A central role for microbial flavohemoglobin. *Free Radical Biology and Medicine* **52**, 1620–1633 (2012).
39. Hernández-Urzúa, E. *et al.* Flavohemoglobin Hmp, but not its individual domains, confers protection from respiratory inhibition by nitric oxide in Escherichia coli. *Journal of Biological Chemistry* **278**, 34975–34982 (2003).
